# Cytokinin Production by the Rice Blast Fungus Is a Pivotal Requirement for Full Virulence

**DOI:** 10.1371/journal.ppat.1005457

**Published:** 2016-02-22

**Authors:** Emilie Chanclud, Anna Kisiala, Neil R. J Emery, Véronique Chalvon, Aurélie Ducasse, Corinne Romiti-Michel, Antoine Gravot, Thomas Kroj, Jean-Benoit Morel

**Affiliations:** 1 Université Montpellier, UMR BGPI INRA/CIRAD/SupAgro, Montpellier, France; 2 Biology Department, Trent University, Peterborough, Ontario, Canada; 3 Department of Plant Genetics, Physiology and Biotechnology, University of Technology and Life Sciences, Bydgoszcz, Poland; 4 INRA, UMR BGPI INRA/CIRAD/SupAgro, Montpellier, France; 5 Université Rennes 1, UMR1349 IGEPP, Rennes, France; Purdue University, UNITED STATES

## Abstract

Plants produce cytokinin (CK) hormones for controlling key developmental processes like source/sink distribution, cell division or programmed cell-death. Some plant pathogens have been shown to produce CKs but the function of this mimicry production by non-tumor inducing pathogens, has yet to be established. Here we identify a gene required for CK biosynthesis, *CKS1*, in the rice blast fungus *Magnaporthe oryzae*. The fungal-secreted CKs are likely perceived by the plant during infection since the transcriptional regulation of rice CK-responsive genes is altered in plants infected by the mutants in which *CKS1* gene was deleted. Although *cks1* mutants showed normal *in vitro* growth and development, they were severely affected for *in planta* growth and virulence. Moreover, we showed that the *cks1* mutant triggered enhanced induction of plant defenses as manifested by an elevated oxidative burst and expression of defense-related markers. In addition, the contents of sugars and key amino acids for fungal growth were altered in and around the infection site by the *cks1* mutant in a different manner than by the control strain. These results suggest that fungal-derived CKs are key effectors required for dampening host defenses and affecting sugar and amino acid distribution in and around the infection site.

## Author Summary

The role of plant-like hormonal compounds produced by fungal pathogens during infection has not been elucidated. Here we identified a conserved gene in most fungi, required for cytokinin production by the rice blast fungus and for its full virulence. Fungal-derived cytokinins are likely potent inhibitors of plant immunity. They are also needed to maintain elevated sugar contents at the site of infection and to drain or consume essential amino acids at, and around, the infection site. Thus, cytokinins represent the second example, after the bacterially-produced coronatine, of plant hormones hijacked by pathogens to successfully invade plant tissues. These findings also suggest that this invasion strategy could be widely conserved among fungi.

## Introduction

Plant pathogens have evolved sophisticated strategies to manipulate host biological processes during infection [1,2]. (Hemi)biotrophic pathogens produce and secrete effector proteins and metabolites to hijack cellular metabolism of the infected tissues to their own benefit. For instance, the bacterial pathogen *Xanthomonas oryzae pv*. *oryzae* produces Transcription Activator Like effectors that specifically induce the expression of genes coding for sugar transporters and thus enhance bacterial nutrition [3]. The virulence factors can also participate to the inhibition of plant defenses that lead to cell death, thus contributing to maintaining infected cells alive [4]. Plant-pathogen interactions shaped the pathogen virulence arsenal and the host immune response system. A first layer of plant defenses is induced by the perception of pathogen- or microbe-associated molecular patterns, like flagellin from bacteria or chitin from fungi [5]. These basal defense responses consist of an early accumulation of reactive oxygen species (ROS), a thickening of the cell wall, and production of metabolites/enzymes with antimicrobial activities. To limit these defenses triggered by chitin perception by the plant’s chitin receptor, fungal pathogens like *Magnaporthe oryzae* secrete chitin-binding effectors that enable escape from the host recognition system [6]. Pathogens also interfere with other steps of plant immunity like signaling cascades following recognition and transcriptional regulators of host defenses [2,7].

Plant pathogens manipulate components of hormonal pathways, whether the corresponding hormones are involved in disease resistance (i.e. salicylic acid, jasmonic acid and ethylene [8]) or in the control of plant developmental processes (i.e. auxin, cytokinins and gibberellic acid [8–11]). Pathogens can affect hormonal homeostasis by targeting/secreting enzymes involved in hormone metabolism or by producing hormonal/mimicking compounds and thereby inhibit defenses, modify nutrient flows and/or induce symptom development. For instance, the fungal pathogens *Ustilago maydis* and *Magnaporthe oryzae* secrete respectively a chorismate mutase and a monooxygenase affecting salicylic acid or jasmonic acid homeostasis during infection and then contributing to their virulence [12,13]. Plant pathogens can also directly produce hormones or compounds with similar biological activities. The bacterial pathogen *Pseudomonas syringae* produces coronatine to mimic jasmonic acid, which actively opens stomata and counteracts salicylic acid accumulation [14,15]. As a consequence, these combined effects on several processes required for infection facilitate host invasion. In the case of pathogenic fungi, there is, however, no direct evidence that hormonal compounds produced are required for virulence.

Fungi and bacteria produce hormonal compounds that are chemically identical or very close to plant hormones involved in plant developmental processes such as cytokinins (CKs) [16–22]. CKs are adenine derivatives that differ in their side chains (aromatic or isoprenoid). In plants, isoprenoid CKs are mainly synthesized through a *de novo* biosynthesis pathway from adenosine phosphate. In this pathway, Isopentenyl transferase (IPT) enzymes perform the first step of biosynthesis. However, another minor CK biosynthesis pathway involving tRNA modification is also described in plants and yeast. This minor pathway requires tRNA-Isopentenyl Transferases (tRNA-IPT), enzymes that perform tRNA modification leading to CK production after tRNA degradation [23–25]. In plants, CKs were originally studied for their effects on cell division/differentiation [26]. CKs also modulate nutritional source/sink distribution and programmed cell-death processes like xylem differentiation, senescence and hypersensitive response [27–30]. Tumor-forming pathogens are striking examples of microbes which are able to produce CKs, like the fungi *U*. *maydis* [31] and *Claviceps purpurea* [32] or the protist *Plasmodiophora brassicae* [33]. Morrison et al., (2015a) [31] have recently shown that CK accumulation in *U*. *maydis* infected tissues is correlated to the virulence of this pathogen. However, in this latter case the evidence that these hormones are produced by the pathogen is still unclear. Likewise, the pathogenic bacterium *Rhodococcus fascians* secretes CKs to interfere with host CK signaling pathways by affecting the transcriptional regulation of key cell cycle genes leading to tumor development [34]. In addition to having potential roles in the virulence of tumor-inducing pathogens, CKs have long been suspected to participate to virulence of pathogens that do not trigger tumors or organ deformations. However, it has not been demonstrated that the CKs produced by this type of pathogens are key virulence factors.

Because CKs delay plant senescence by limiting oxidative burst and maintaining photosynthesis activity [35], they are at the cross-road of several pathways of interest for manipulation by pathogens that need to keep host cells alive in order to drain nutrients for their own growth. Consistent with this role, CKs are accumulated in “green islands”, which are tissues corresponding to photosynthetically active zones maintained around pathogenic lesions caused by (hemi)biotrophic pathogens [36–39]. CK compounds have been measured in several different fungal species (even in non-plant interacting ones [40]) and CK secretion was mostly observed in the case of (hemi)biotrophic microbes [32,41]. For instance, Hu & Rijkenberg (1998) immuno-detected CKs in and around fungal hyphae of *Puccinia recondita* f.sp. *tritici* during wheat infection [42].

The hemibiotrophic fungus *Magnaporthe oryzae* responsible for the rice blast disease, also produces CKs *in vitro* [43] but the CK biosynthesis pathway in this fungus has not been established. Moreover, the role of CKs in the virulence of filamentous fungi that, like *Magnaporthe*, do not form tumors has never been demonstrated. In this study we identified a gene coding for a putative tRNA-Isopentenyl Transferase (tRNA-IPT) in the genome of *M*. *oryzae* and in all Ascomycete genomes tested. Mutants deleted for this gene, named *Cytokinin Synthesis 1* (*CKS1*), were generated and were found to be impaired in CK production confirming that the tRNA degradation pathway is involved in fungal CK production in *Magnaporthe* as was suggested for other fungi by Morrison et al., (2015b) [40]. Deleted *cks1* mutants were not affected in their *in vitro* growth or asexual development in standard minimal conditions however they showed severely reduced virulence on rice. Remarkably, the CK-deficient mutant was not able to maintain the levels of several key nutrients at the infection site and induced early and strong plant defenses, suggesting that fungal CKs may contribute to metabolite mobilization and to rice defense inhibition. Our work confirms the view that, in *M*. *oryzae*, and possibly in many other fungi, this putative tRNA-IPT, contributes to CK production. Since *CKS1* is required for the full virulence of *Magnaporthe*, we propose that CKs from fungal pathogens could act as effectors combining functions in defense inhibition and nutrient mobilization.

## Results

### *In silico* analysis of *M*. *oryzae* genome revealed putative orthologous genes of yeast and plant cytokinin pathways

To investigate the role of CKs in the virulence of the blast fungus *M*. *oryzae*, homologs of plant and yeast genes involved in CK metabolism or signaling were searched in the *M*. *oryzae* genome using BLASTp. Potential orthologous genes of some important CK biosynthesis, degradation and signaling component coding genes were identified (S1 Table), further extending previous reports that the CK signaling and metabolic pathways are present in *M*. *oryzae*. As previously mentioned, independently of the *de novo* biosynthesis pathway, CKs can be also produced through tRNA degradation. In this second pathway the first step of CK production involves tRNA-Isopentenyl transferases (tRNA-IPT), as previously described in *A*. *thaliana* (AtIPT2 and AtIPT9) and *Saccharomyces cerevisiae* (MOD5) [23–25,44,45]. A putative tRNA-IPT protein, coded by MGG_04857, was identified in *M*. *oryzae* which shared 36% identity with AtIPT2 or MOD5 and the sequences of the different known interaction sites were highly conserved (S1A Fig). No other gene containing the coding sequence of the IPT domain was identified, suggesting that there is only one orthologous gene in *M*. *oryzae*. We modeled the three-dimensional structure of MGG_04857 (S1B Fig) by protein threading, a method based on fold recognition [46] and using the experimentally determined MOD5 structure as a template. The model produced suggested a conserved structure, but also confirmed a high conservation of primary sequences of interaction sites i.e. the ATP, DMAPP as well as tRNA binding sites (S1B Fig [47,48]) and indicated that MGG_04857 shows all the features of a *bona fide* tRNA-IPT. Taken together, these results support the hypothesis that the blast fungus can produce CKs [43], potentially perceive them, and suggest that MGG_04857 could act as a key enzyme in fungal CK production.

### Production of *MGG_04857 (CKS1- Cytokinin Synthesis 1)* mutants and their growth *in vitro*

To test the involvement of the putative tRNA-IPT coded by the *MGG_04857* gene in fungal CK production, knock-out mutants of this gene, later called *cks1* (see below), were generated by homologous recombination in the *M*. *oryzae* GY11 genetic background (S2 Fig). In addition, a complemented strain (*cks1* ^*CKS1*^) was built by transformation of the *cks1* mutant strain with a construct carrying the genomic sequence of *MGG_04857* under control of its own promoter. Gene disruption and complementation were confirmed by PCR on genomic DNA and qRT-PCR and showed that the expression of *MGG_04857* was similar between wild type and *cks1* ^*CKS1*^ isolates, but not detected in the *cks1* mutants (S2E Fig).

Since yeast MOD5 mutants are known to display altered phenotypes [49], we measured the growth of *cks1* mutants under normal and environmental stress. The *in vitro* growth of the *cks1* mutant was not different from the wild type or *cks1* ^*CKS1*^ strains under standard minimal growth conditions (S3A Fig). Moreover, the development of infection structures like the appressorium was not impaired by the *cks1* mutation on glass slides (S3B Fig) or on rice leaf surface (S3C Fig). This suggests that the early steps of fungal growth are not significantly modified by the *cks1* mutation. By contrast, the *cks1* mutant showed slight but significant and reproducible reduced growth when grown under 1mM H_2_O_2_ (S4A Fig). This phenotype could be complemented by the addition of the CK kinetin to the medium (S4B Fig), suggesting that this defect could be related to a reduced CK production due to the *MGG_04857* deletion.

### *MGG_04857 (CKS1*- *Cytokinin Synthesis 1)* is required for CK biosynthesis in *M*. *oryzae*

To investigate the role of MGG_04857 in CK production, CK levels were determined by liquid chromatography-positive electrospray ionization tandem mass spectrometry (LC(ESI+)-MS/MS) [19,50] both in the culture supernatant and mycelia of the different strains (Table 1).

**Table 1:** The *cks1* mutant does not produce cytokinins.

	Mycelia (pmol/g FW)				Supernatant (pmol/30mL)			
	Nucleotide forms		Riboside forms		Nucleotide forms		Riboside forms	
	cisZNT	iPNT	cisZR	iPR	cisZNT	iPNT	cisZR	iPR
*cks1*	0.0 +/- 0.0	0.0 +/- 0.0	0.0 +/- 0.0	0.0 +/- 0.0	0.0 +/- 0.0	0.0 +/- 0.0	0.0 +/- 0.0	0.0 +/- 0.0
*cks1* ^*CKS1*^	10.7 +/- 1.5	0.4 +/- 0.3	216 +/- 45.1	13.2 +/- 6.1	16.7 +/- 3.2	1.1+/- 0.1	1.7 +/- 0.5	0.4 +/- 0.1
GY11	7.8 +/- 5.3	0.7 +/- 0.2	164 +/- 126	13.0 +/- 9.9	14.8 +/- 11.9	1.2 +/- 0.5	2.9 +/- 1.7	1.1 +/- 0.3

CKs quantification was performed by liquid chromatography-positive electrospray ionization tandem mass spectrometry. *cks1* corresponds to mutant deleted for MG_04857, *cks1* ^*CKS1*^ to the complemented strain and GY11 to the original wild type strain.

The CKs detected were: cisZNT (cis-zeatin nucleotide), iPNT (isopentenyladenine), cisZR (cis-zeatin riboside) and iPR (isopentenyladenosine). For each strain, four replicates were analyzed; the mean and SD are indicated. This experiment was repeated twice with similar results.

Four major isoprenoid CKs, cisZR (*cis*-zeatin riboside), iPR (isopentenyl adenosine), cisZNT (*cis*-zeatin nucleotide) and iPNT (isopentenyladenine nucleotide) were detected in mycelia and supernatant of the wild type GY11. The riboside forms, cisZR and iPR, were the most abundant CKs in mycelia while the nucleotide precursor forms, cisZNT and iPNT, were the main secreted ones. The other CK forms, trans-zeatin and dihydrozeatin were not detected. Thus, in the minimal medium, cisZR and cisZNT are the major CKs produced and secreted by the wild-type strain. This is consistent with CK measurements made on several other fungi where cis-zeatin forms were the main CKs detected [40]. The *cks1* mutant was not able to produce and/or secrete any detectable CKs whereas the complemented strain, *cks1* ^*CKS1*^, produced CKs at similar levels as the wild type strain GY11 did. Thus, *MGG_04857* appears to be required for CK biosynthesis in the rice blast fungus (therefore called *CKS1* for *Cytokinin Synthesis 1*) and is likely coding for an active tRNA-IPT protein, although this activity needs to be established.

### *M*. *oryzae cks1* mutants have reduced virulence on rice

**Fig 1:**
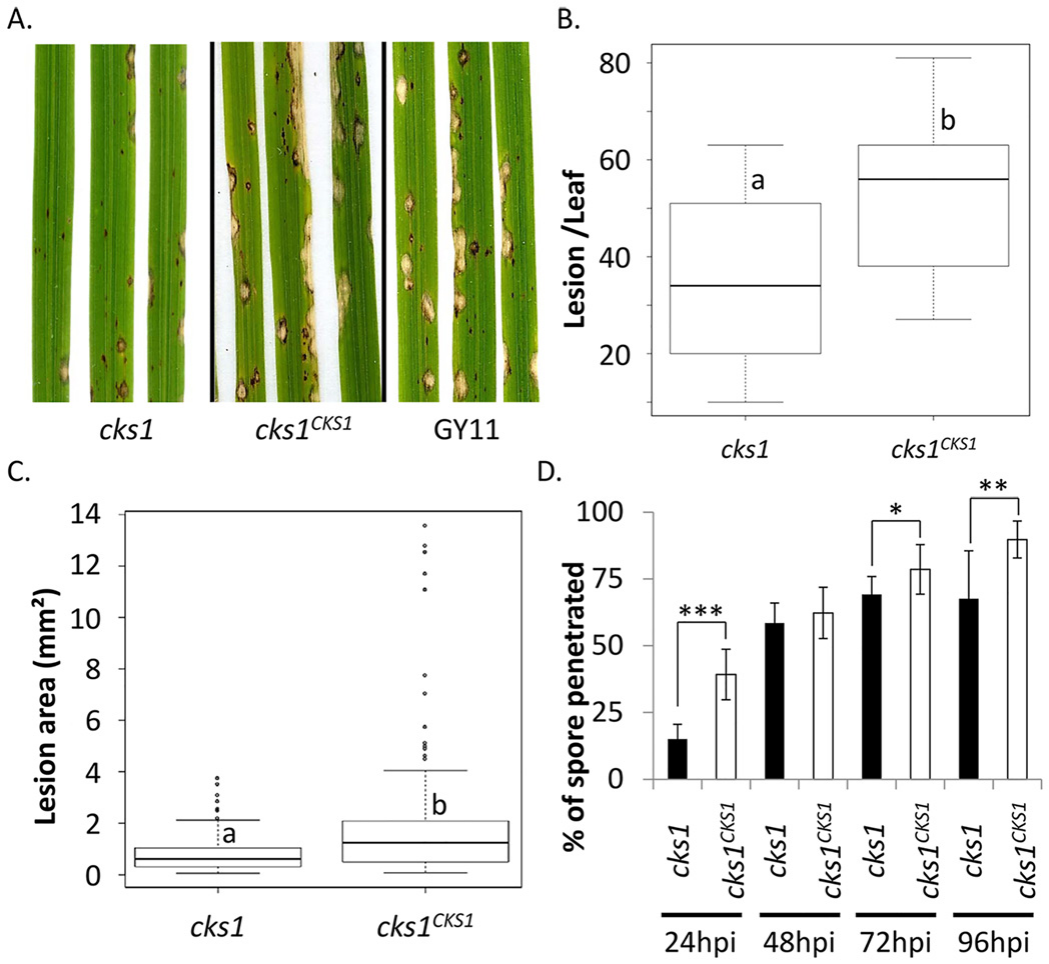
The *cks1* mutant strain is less virulent than wild-type and complemented control strains. Nipponbare plants were inoculated with *Magnaporthe cks1* mutants and control strains to evaluate virulence. The results for control GY11 and the complemented strain were similar and are only shown for symptoms. (A) Disease symptoms were observed 6 days after inoculation. Grey spots represent susceptible lesions whereas brown spots represent failed penetration events. The number of susceptible lesions per leaf (generalized linear model, p-value = 0.02) and size of lesions (mixt model, p-value = 0.003) were measured as shown in (B) and (C) respectively (for more details see materials & methods). The values represent the mean and SD from four biological replicates each composed of 6 plants. The percentage of spores from the *cks1* and *cks1* ^*CKS1*^ that penetrated the leaf was measured (D) under the microscope at different time points (hpi: hour post-inoculation). The data presented is the mean and SD of three biological replicates (>100 infection sites/replicate). A t-test was used to compare the penetration of the mutant and control strains, *, p-value < 0.01; **, p-value < 0.002; ***, p-value < 3.10^−5^. All experiments were repeated three times with similar results and one representative experiment is shown here.

To test for involvement of fungal CKs in the interaction between rice and the blast fungus, we inoculated mutant and control strains on the susceptible rice cultivar Nipponbare. The *cks1* mutant strain was less virulent than *cks1* ^*CKS1*^ or GY11 wild-type strains as shown by a reduction of disease symptoms (Fig 1A).

The CK-deficient strain produced less grayish, sporulating lesions per leaf (Fig 1B) and these lesions were smaller than those caused by control strains (Fig 1C). These results suggest that leaf penetration (reflected by lesion number) and invasion (reflected by lesion size) of the CK-deficient strain are both impaired, although not completely abolished. Impaired penetration was confirmed by microscopic observation of individual interaction sites which showed that *cks1* failed to penetrate as efficiently as *cks1* ^*CKS1*^ during the early times of infection (<24 h, Fig 1D). This is likely due to defective steps after appressorium formation since this developmental stage was unaffected by the *cks1* mutation (S3C Fig).

### The reduced virulence of *cks1* mutant is linked to the absence of cytokinins

**Fig 2:**
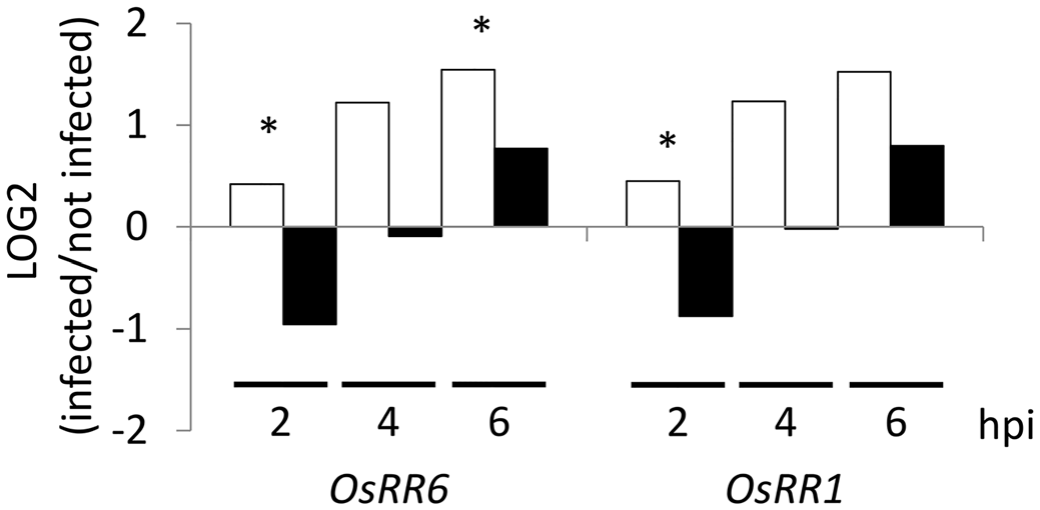
Plant cytokinin signaling is differently affected during *cks1* infection. The transcriptional regulation of CK marker genes (*OsRR6* (Os04g57720) and *OsRR1* (Os11g04720) as named by Pareek et al., (2006)[53] was evaluated by quantitative RT-PCR using the Actin gene for normalization. Nipponbare plants were inoculated with spore suspension (in gelatin 0.5%) of either the *cks1* mutant (black bars) or *cks1* ^*CKS1*^ control strain (white bars) and gene expression was measured at 2, 4 and 6 hours post inoculation (hpi), before penetration of the leaf tissues but at stages where there was no significant difference in growth of the *cks1* mutant compared to the complemented strain (S3C Fig). The values presented are the Log2 ratios (infected/not infected) of the means calculated from four independent replicates. Uninfected plants were sprayed with gelatin 0.5% but without spore suspension. This experiment was repeated three times and showed similar results. A t-test was used to compare the means of expression quantified in *cks1* (black bars) and *cks1* ^*CKS1*^ (white bars) inoculated plants. *: values significantly different at p-value < 0.05.

As a test for biological activity of fungal-produced CKs, we performed qRT-PCR and quantified the expression of rice Response Regulator (RR) genes *OsRR6* and *OsRR1* (Fig 2) that were previously described to be transcriptionally regulated by CKs [51,52] and can thus be used as CK bio-sensors [43].

During early contact of fungal conidial spores with the plant surface (2h, 4h, 6h), and at a stage where all strains showed similar growth *in vitro* or on the plant surface (S3C Fig), the two CK response markers tested had significantly lower expression in plants inoculated with *cks1* than with *cks1* ^*CKS1*^ strains. Similar results were found for all other *RR* genes tested (S5A Fig). This result supports the hypothesis that fungal CKs affect host CK signaling pathway and is consistent with the report made by Jiang et al., (2013) [43] that conidia, the first *M*. *oryzae* cells in contact with the host, can produce CKs.

**Fig 3:**
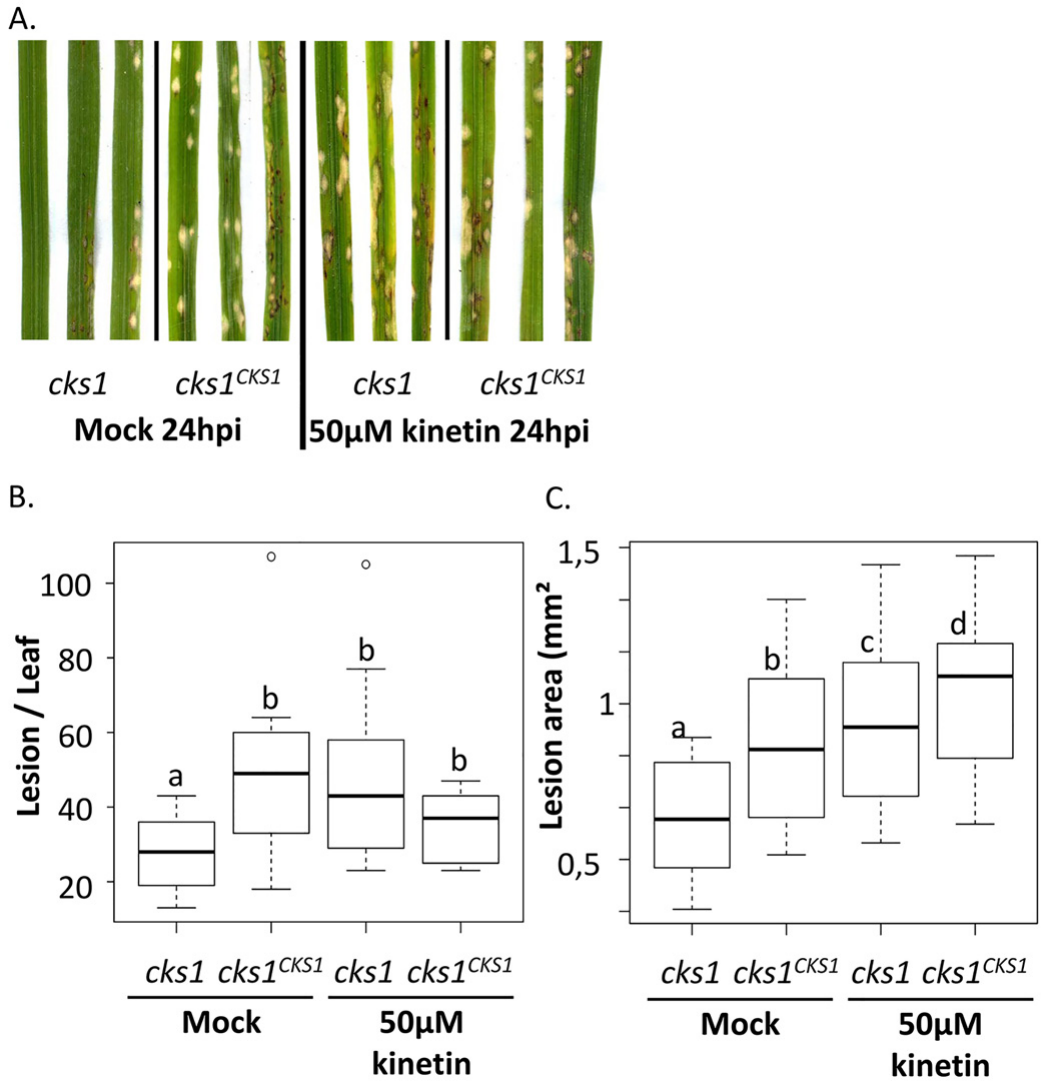
The virulence of the *cks1* strain is fully restored by an exogenous application of cytokinin. One day after inoculation with the *cks1* mutant or the *cks1* ^*CKS1*^ control strain, plants were treated with 50 μM of the CK compound, kinetin, or buffer alone. Kinetin alone without infection had no visible effect on leaf aspect. (A) The symptoms were observed 6 dpi. The number of lesion per leaf (B) and the size of lesions (C) were measured. The values represent the mean and SD of three biological replicates of 10 individuals. The entire experiment was repeated 3 times with similar results. The different letters indicate significant differences between values (p-value < 0.01) as estimated by a generalized linear model (B) or a mixed model (C) using ANOVA analysis (See Materials and Methods).

To test if CKs could restore the virulence of the mutant strain, we exogenously applied the CK kinetin at 24h after inoculation (Fig 3) since the delay of penetration of the mutant is noticed at this time (Fig 1D). Kinetin was applied in the same conditions as previously described [43]. Kinetin treatment fully restored the virulence of *cks1* since the number and the size of lesions caused by *cks1* were similar to those caused by the *cks1* ^*CKS1*^ complemented strain (Fig 3). Similar results were obtained with an exogenous application of cis-zeatin (S6 Fig), which is the major cytokinin produced by *M*. *oryzae* (92% of CKs in supernatant; Table 1). These data strongly support the hypothesis that the reduced virulence of the *cks1* mutant is directly due to a defect in CK production.

### The *cks1* mutant induces a stronger oxidative burst than control strains

**Fig 4:**
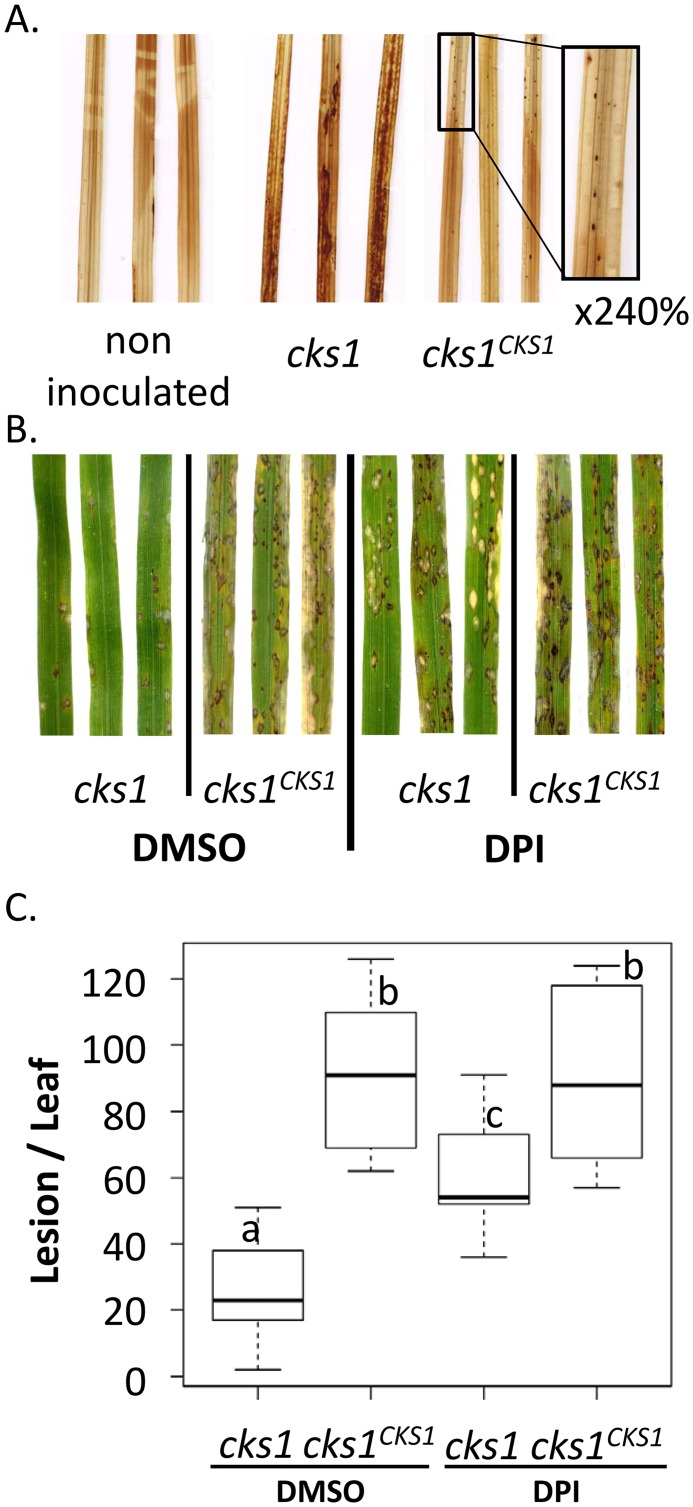
The impaired virulence of *cks1* correlates with an enhanced induction of the oxidative burst and can be partially restored by inhibiting NAD(P)H oxidase activity. The relationship between virulence and reactive oxygen species accumulation was evaluated in the *cks1-*infected leaves. (A) The oxidative burst was detected 48 h after inoculation using DAB stain that turns brown upon reaction with H_2_O_2_. Brown spots correspond to sites where the wild-type blast fungus penetrated (see inlet), whereas a global browning was visible with infection with *cks1* mutant. This experiment was repeated two times and gave similar results. (B, C) DPI, an NAD(P)H oxidase inhibitor partially restores the virulence of the *cks1* mutant. One day after inoculation (once appressorium formation was initiated), plants were treated with DPI (0.5μM diluted in DMSO as previously described [54]. The symptoms were observed 6 dpi (B) and the number of lesions per leaf was measured (C). The letters indicate significantly different values according to a generalized linear model and ANOVA analysis (p-value < 0.04), see Materials and Methods.

To evaluate the effect of fungal-derived CKs on early host basal defense responses, we measured ROS accumulation. Compared to *cks1* ^*CKS1*^, the accumulation of H_2_O_2_ (as revealed by DAB staining) was much more pronounced in response to the *cks1* mutant (Fig 4A) and extended well beyond penetration sites (punctuated brown spots in complemented mutant controls) since the DAB staining was visible throughout all leaf tissues.

In order to test if the *cks1* mutant was able to infect the host more efficiently if ROS production was impaired, we treated inoculated plants with DPI (Diphenylene Iodonium), an inhibitor of flavor-enzymes like the NAD(P)H oxidase involved in H_2_O_2_ production [55]. The virulence of *cks1* mutant was partially restored when plants were treated with DPI (Fig 4B and 4C). This suggests that the capacity of the *cks1* mutant to invade the host cell is restored, although only partially, when ROS production is reduced.

### The *cks1* mutant induces an early and strong transcriptional response of rice defense markers

**Fig 5:**
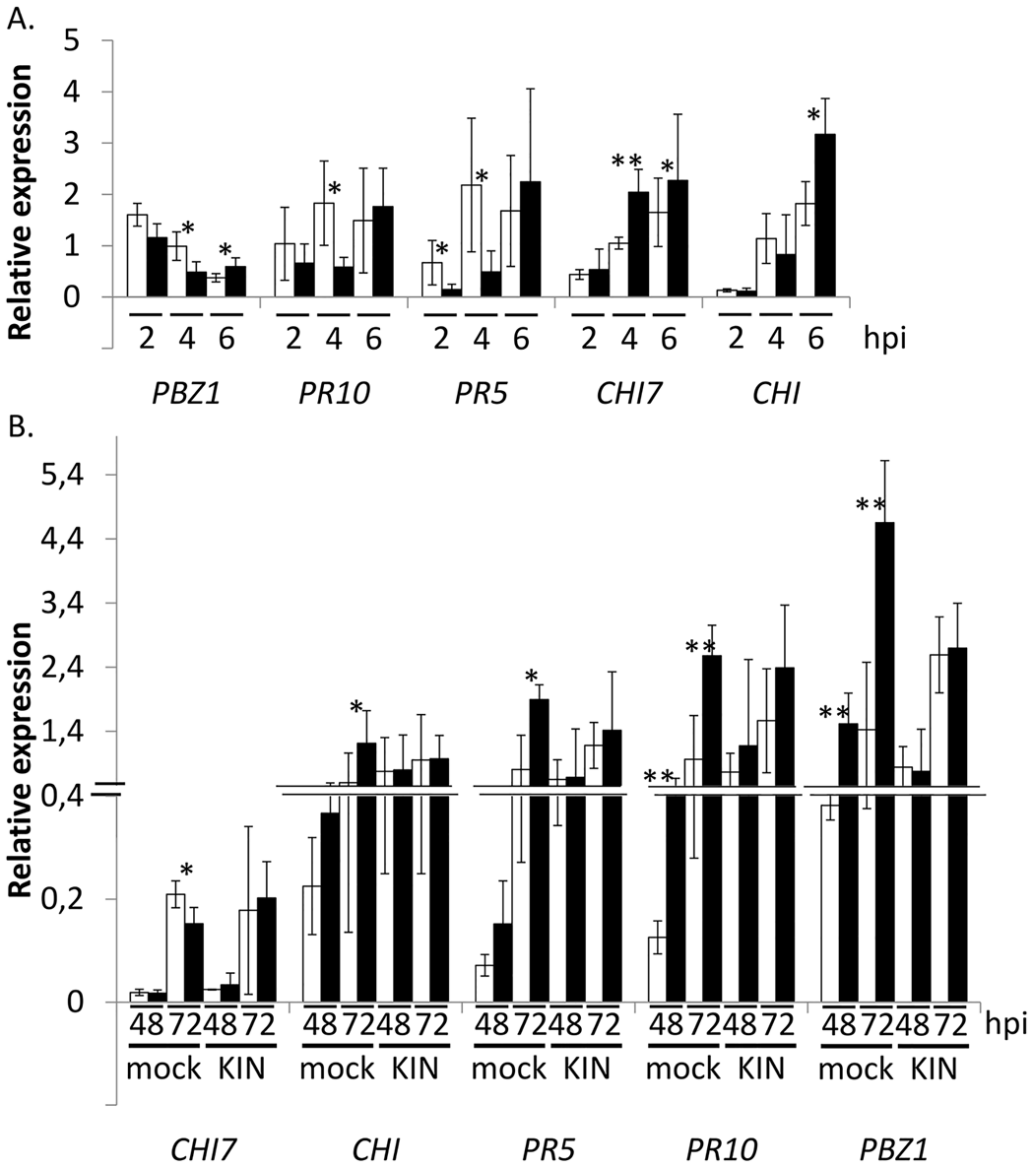
The impaired virulence of *cks1* correlates with enhanced induction of defense genes and can be partially restored by exogenous cytokinin. The transcriptional regulation of defense-marker genes was evaluated upon inoculation with *cks1* mutant and complemented control strain. Nipponbare plants were inoculated with spore suspension (in gelatin 0.5%) of either the *cks1* mutant or *cks1* ^*CKS1*^ control strain. Gene expression (normalized by plant Actin gene) was measured at different times after inoculation. *PBZ1* is a classical disease-related marker coding for a PR10 protein [56], *PR5* and *PR10* are classical disease-related markers [57], *CHI* and *CHI7* are chitinases [58]. (A) Gene expression was measured before the first indications of fungal penetration (< 6hpi). (B) Kinetin (50 μM) was applied (KIN) or not (mock) at 24 hpi and gene expression was also measured at 48 hpi. A t-test was used to compare the means between *cks1* and *cks1* ^*CKS1*^; for one given gene * indicate significant differences between *cks1* and complemented strain; for (A) *, p-value < 0.04 and **, p-value<0.002 and for (B) *, pvalue<0.03; **, p-value<0.008. The values presented are the means calculated from four independent replicates. The experiments were repeated twice with similar results.

We measured the expression of some well-established rice defense-marker genes during infection with the different strains. It first appeared that the defense-marker genes were differentially expressed earlier (2 to 6 hpi; Fig 5A and S5B Fig) in plants infected with *cks1*—before fungal penetration that mostly occurs after 24h (Fig 1D). After penetration of the fungus (>24 hpi) and very strikingly, many defense-markers tested also showed a stronger induction in plants infected with *cks1* as compared to those infected with the control *cks1* ^*CKS1*^ (e.g. *CHI*, *PBZ1*, *PR10* and *PR5* in Fig 5B).

In the next step, we tested whether this over-induction of defense-markers by *cks1* was affected when the *cks1* virulence was complemented by exogenous application of the CK kinetin. This was the case for most genes that initially showed an over-induction (e.g. *CHI*, *PBZ1* and *PR5*; Fig 5B). It indicates that complementation by exogenous cytokinin treatment reverts penetration and growth of the fungus as well as plant it brings back defense-marker expression to normal levels. Altogether these results support the hypothesis that the loss of virulence and the over-induction of defense associated with the *cks1* mutation are linked to an absence of CK production by the mutant strain.

### The virulence of the *cks1* mutant can be restored in immuno-depressed plants

The enhanced oxidative burst and defense-genes expression is consistent with the reduced virulence phenotype observed with the *cks1* mutant. This suggests that fungal CKs normally produced by *M*. *oryzae* may inhibit key plant defense reactions. To further support this hypothesis, we tested the capacity of the *cks1* isolate to infect rice mutants which are defective for the chitin receptor, CEBiP, and the master transcriptional regulator NH1 that are both known to be immuno-depressed [57,59].

**Fig 6:**
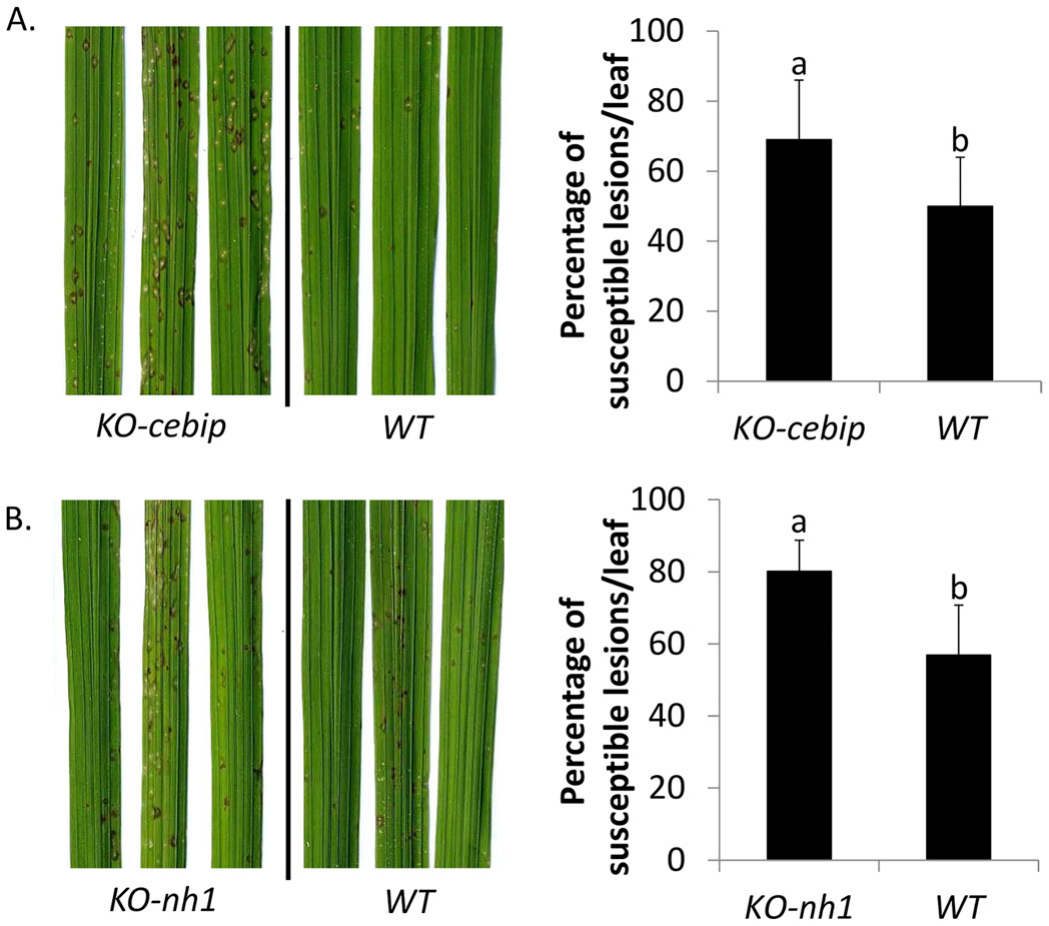
The virulence of *cks1* can be restored in immuno-deficient rice mutants. The *cks1* strain is more virulent on rice mutants deficient for basal defenses. KO-*cebip* (A) and *KO-nh1* (B) rice mutants and control plants (WT) [57], all in Nipponbare genetic background, were spray-inoculated with *cks1*. Symptoms were measured 6 days after inoculation on three replicates containing 6 plants. The values are the mean and SD from three biological replicates. A t-test was used to compare the percentage of susceptible lesions of *cks1* on immune-deficient mutant and respective control plants, p-value < 0.02.

The fungal *cks1* mutant was more virulent on *cebip* and *nh1* rice mutants than on the wild-type plants (Fig 6). The restoration of virulence of the *cks1* mutant on rice mutant plants impaired for defense responses strongly supports the hypothesis that the *cks1* mutants have the capacity to infect rice as long as defenses are inhibited.

### Elevated fertilization can restore virulence of the *cks1* mutant

**Fig 7:**
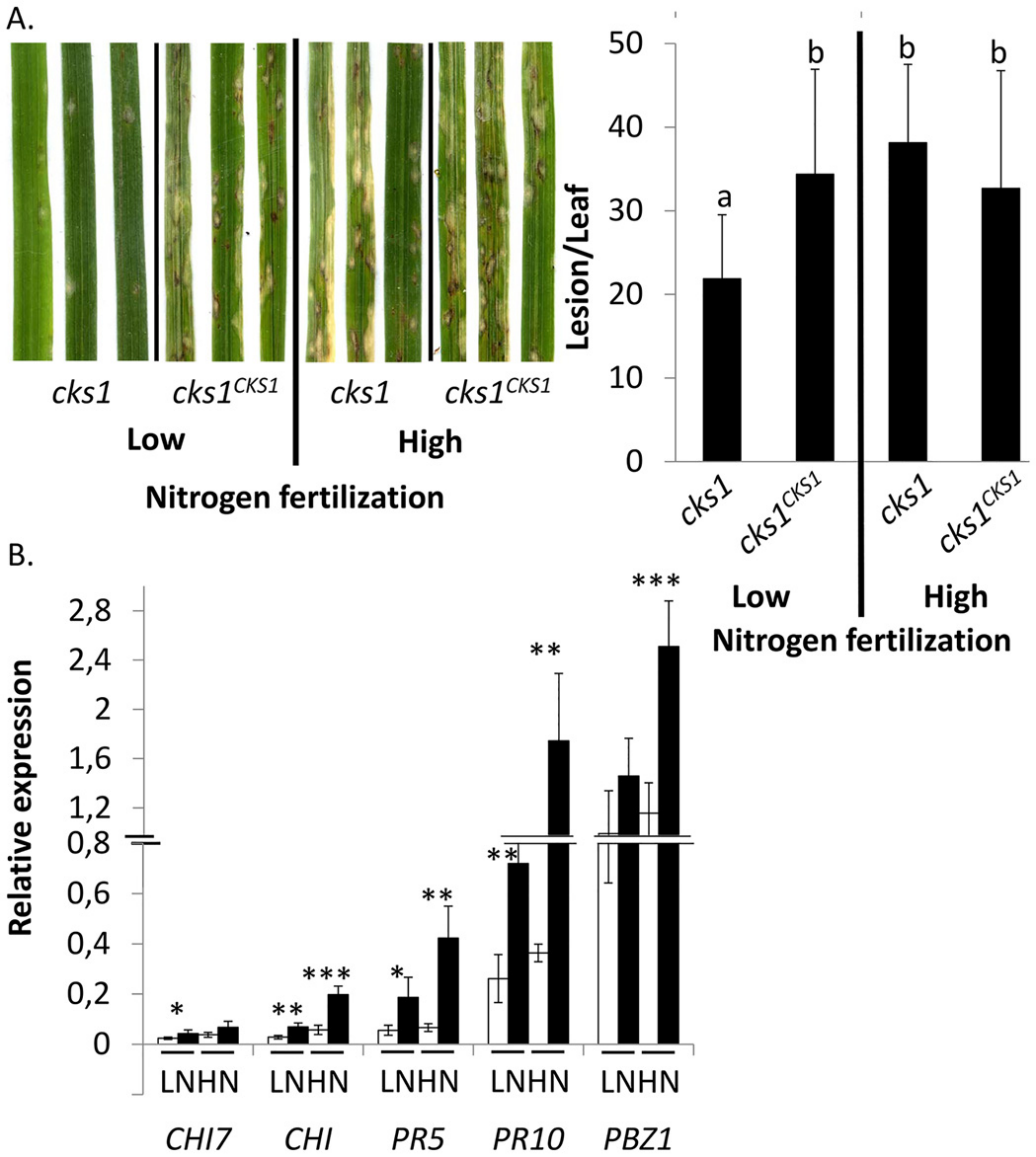
High fertilization levels restored *cks1* virulence without inhibiting defense induction. Plants were fertilized (high fertilization) or not (low fertilization) 24h before inoculation. Fertilization was done as in Ballini et *al*, 2013 [61] to test the effect of plant nutritional status on *cks1* virulence. (A) Symptoms 6 days after inoculation and the number of lesion per leaf in plants inoculated with *cks1* mutant or *cks1* ^*CKS1*^ under low or high nitrogen fertilization. Three biological replicates composed of 10 plants were analyzed per strain/condition. The different letters indicate significant differences between values (p-value < 0.03) as estimated by a t-test. (B) The expression of defense-marker genes was measured 48hpi and first normalized with *Actin* in Nipponbare plants inoculated with *cks1* ^*CKS1*^ (white bars) or with *cks1* (black bars), fertilized (High Nitrogen, HN) or not (Low Nitrogen, LN) 24h before inoculation. For each gene, the mean and the SD of relative expression obtained from 4 biological replicates (each of 3 plants) are presented. A t-test was done on raw data to compare relative expression in *cks1* and *cks1* ^*CKS1*^ inoculated plants. *, p-value<0.04; **, p-value<0.003; ***, p-value<0.0005.

CKs affect different key metabolic processes in plants, like Calvin-Benson or tricarboxylic acid cycles and mediate source/sink modifications [29]. Therefore, we hypothesized that the lack of virulence of the CK-deficient strain could be also due to a reduced capacity to exploit or drain nutrient resources. Under this hypothesis, we reasoned that high levels of fertilization could enhance *cks1* virulence. Indeed high fertilization levels were previously shown to increase amino-acid and sugar contents in rice leaves [60] as well as rice blast susceptibility [61]. The protocol described in [61] was used to fertilize plants 24h before inoculation with *cks1* (Fig 7). Under high fertilization regime, plants were more susceptible to the *cks1* mutant (Fig 7A), supporting the hypothesis that this mutant is able to infect rice tissues but likely requires external complementation with essential nutrients.

We then evaluated if the over-induction of defense-markers, observed in plants infected with the *cks1* mutant (Fig 5B), was still visible when the virulence was reversed by fertilization. Quite remarkably, the over-induction of defense-marker genes tested did not revert under high fertilization (Fig 7B) in *cks1*-infected plants, despite the fact that the virulence of this mutant strain was restored (Fig 7A). The data strongly support the idea that the over-induction of defense-markers by *cks1* is not due to an arrest of growth itself (which would subsequently trigger enhanced plant defense) but rather to another defect that leads to reduced virulence, likely in CK production. The virulence of the *cks1* mutant despite high expression of defense is likely compensated by high nutrient availability under high fertilization.

### The *cks1* mutation affects sugar and amino acid contents in and around rice infected tissues

**Fig 8:**
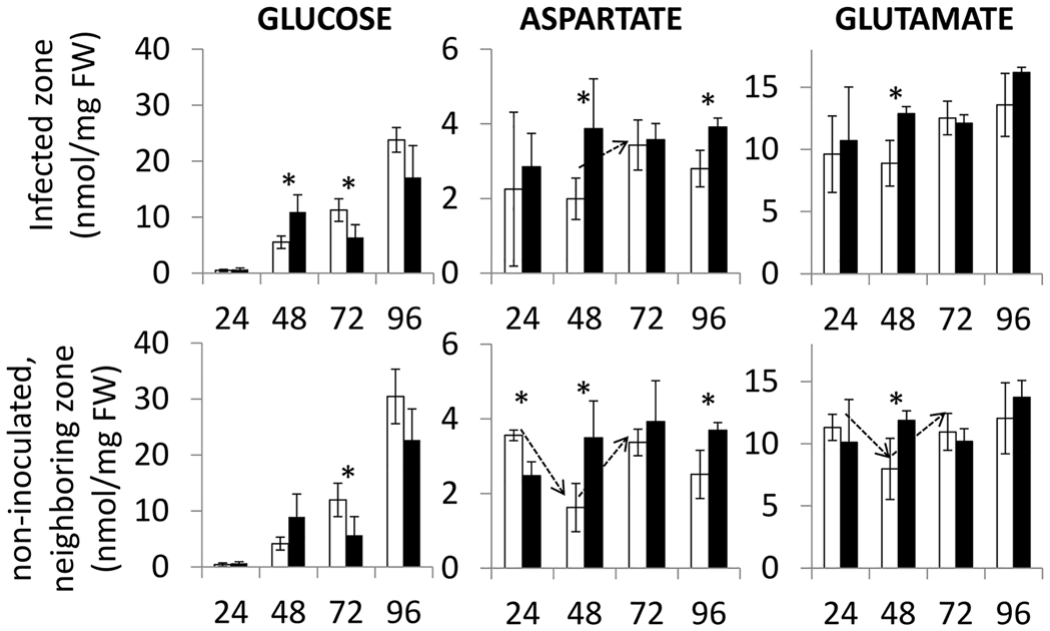
The impaired virulence of *cks1* is associated with altered contents of nutritional elements essential for the fungus. Metabolomic analysis of plants infected with *cks1* and complemented mutant strains. Glucose, aspartate and glutamate contents were quantified (nmol/mg of fresh weight) as well as other sugars and amino acids, during infection (times are indicated), at the site of inoculation corresponding to the “infected zone” and one centimeter apart on both sides (upper and lower) with respect to the inoculated zone (S7 Fig). Only the lower “non-inoculated, neighboring part of the leaf” is shown but all data relative to the “upper non-infected zone” can be found in S7 Fig. For more details see also Materials and Methods. A t-test (*, p-value < 0.05) was used to compare amino acid contents in leaf fragments from plants inoculated with the *cks1* (black bars) and *cks1* ^*cks1*^ control complemented strain (white bars). The dashed arrows point to the significant changes (*t-test, p-value < 0.05) of amino-acid contents in *cks1*-infected plants between 24 and 48 hpi. For each time point four replicates composed of three leaf fragments were analyzed, mean and SD are indicated.

Previous metabolomic analysis of plants infected by the rice blast fungus showed that some amino acids (glutamate and aspartate) as well as sugars (fructose and glucose) are nutrients which are drained towards the infection site [62]. We therefore tested whether *CKS1* is required to efficiently maintain nutrient levels and/or modify nutrient fluxes at and around the penetration site. In order to test this hypothesis, after local deposition of a droplet of spore suspension of the different strains on the leaf surface, we measured the levels of different sugars and amino acids in the blast-infected and the neighboring non-infected zones (Fig 8 and S7 Fig).

During the infection by the *cks* ^*CKS1*^ control strain, glucose (Fig 8A) and fructose (S7 Fig) contents progressively increased in and around the infected zone, which is consistent with previous observations that the accumulation of sugar is associated with successful pathogen invasion [63]. Initially, glucose and fructose contents were significantly higher 48 hpi in plants infected with the *cks1* mutant compared to plants infected with the control strain, an observation that can be related to enhanced induction of defense (see Discussion). By contrast, at later time points (after 48hpi), soluble sugars contents were significantly lower in plants infected with the *cks1* mutant. Given the known effects of CKs on maintaining photosynthesis active [64], this observation supports the idea that CKs produced by *Magnaporthe* could contribute to maintain sugar production during infection.

For most amino acids, there were no strong differences between the tissues infected by *cks1* mutant and those infected by *cks1* ^*CKS1*^ except for aspartate and glutamate (Fig 8A). Away from the infected zone, the concentration of these two amino acids transiently decreased at 48hpi in plants infected with the control strain and increased at the site of infection at 72 hpi, suggesting that these amino acids can be drained towards the infection site or accumulated. By contrast, their level remained almost stable during infection with the *cks1* mutant. This suggests that the *cks1* mutant strain is not able to drain or consume aspartate and glutamate as efficiently as the control strain during infection.

## Discussion

### Identification of *CKS1*, a conserved gene required for fungal cytokinin production

The pathogenic fungus *Magnaporthe oryzae* produces and secretes CKs [43] but its biosynthesis pathway had remained unknown. Moreover, the involvement of CKs in virulence of pathogenic fungi that do not induce tumors was still undetermined. Recently, a cluster including two genes (including one coding for a IPT-LOG) involved in the *de novo* CK biosynthesis pathway, was characterized in the ergot fungus *Claviceps purpurea* [32]. In mutants deleted for these two genes, CK production was partially affected but virulence was not. In the present study, we identified a gene in the rice blast fungus, *CKS1*, required for CK biosynthesis. The protein encoded by this gene presents all the features of a tRNA-IPT enzyme, the type of which is known in plants and yeast, and suspected in many fungi, to perform the first step of one of the CK biosynthesis pathways [25,40,45]. Phylogenetic analysis of tRNA*-*IPT protein sequences suggests that this gene is highly conserved among Ascomycete fungi (S8 Fig) and beyond [47,65]. Our work sets the basis for functional analysis of this pathway in several other plant associated fungi known to produce CKs [41,66,67].

We generated a *cks1* mutant strain and demonstrated that this strain does not produce any of the CK types secreted by the wild type GY11 and *cks1* ^*CKS1*^ complemented strain (Table 1). Moreover, the CKS1 protein is the only one found to contain an IPT domain in the rice blast fungal genome (S1 Table). These results suggest that the CK biosynthesis pathway controlled by CKS1 is probably the only one in the rice blast fungus. Nucleotide forms, which are known to be precursors to riboside, free base and glycosylated CKs [44], seem to be the major type of CKs secreted by control strains (*cks1* ^*CKS1*^ and GY11). This contrasts with the yeast *Δmod5* mutant which was found to still produce CKs, and suggested that tRNA turnover is not mainly involved in yeast CK production [68]. However, the *Δmod5* mutant was grown on medium composed of yeast extract that already contains hormonal compounds. Thus, the free CK production by yeast observed in that study could have come from the recycling of CK compounds provided by the medium.

Like the yeast *Δmod5* mutant, the *M*. *oryzae cks1* mutant had no obvious pleiotropic effects under standard growth conditions in minimal medium (S3 Fig). By contrast, the growth of *cks1* was affected under oxidative stress and this could be reverted by exogenous CKs (S4 Fig). This result suggests that CKs play a role in fungal processes, like in yeast, for which MOD5 has primary roles in translation and is required for antifungal drug resistance [49]. These processes may participate to the loss of virulence of the *cks1* mutants (see below).

In plants, CK signaling is mediated by a multistep phosphorelay system involving Histidine Kinase Receptors, Histidine Phospho-transfer proteins and Response Regulators [53,69]. This kind of transduction system is widespread among organisms [70–73]. Several studies mentioned its involvement in osmoregulation in yeast and in hyphal growth of *Neurospora crassa* [74]. Based on protein sequence homology, putative orthologous genes to those of the plant CK signaling pathway were found in the *M*. *oryzae* genome (S1 Table). Two of these genes, *MoSLN1* (coding for an histidine kinase receptor) and *MoSSK1* (coding for type-A response regulator), were previously shown to be required for full virulence of *M*. *oryzae* [75,76]. This suggests that CKs could also be perceived by the blast fungus in order to trigger a signal potentially required for its virulence. However, the involvement of these proteins in CKs perception and/or in CKs signaling transduction in response to plant or fungal-derived CKs remains to be established.

### Cytokinins are required for fungal virulence

In addition to becoming deficient in CK production, the *Magnaporthe cks1* mutant was less virulent than wild-type GY11 or *cks1* ^*CKS1*^ control strains since its capacity for penetration and invasion was strongly impaired (Fig 1). Mutations affecting fungal invasion are still scarce in *M*. *oryzae* as most mutations affect appressorium formation and penetration only [77]. Only very recent studies reported the role of protein effectors in virulence of *Magnaporthe* during host invasion [78]. The characterization of the CK deficient *cks1* strain demonstrates that CKs play a key role in virulence of the rice blast fungus. Testing whether CKs play similar roles in other pathogenic fungi is now possible since *CKS1* homologs exist in most of them (S8 Fig).

An exogenous supply of kinetin (Fig 3) or cis-zeatin (S6 Fig) post inoculation restored the virulence of the CK-deficient strain, suggesting that the lack of virulence of these mutants was due to their inability to produce CKs. Moreover, exogenous application of kinetin reverted part of the over-induction of defense by the *cks1* mutant (Fig 5B). This suggests that although other defects due to the deletion of the tRNA-IPT gene may exist, they are not responsible for this over-induction of defense. The demonstration that these fungal-derived CKs are secreted *in planta* is technically challenging because of the presence of plant CKs and the difficulty to localize such small metabolites. However the observation that the expression of plant CK responsive genes is differentially affected by the *cks1* strain before penetration in the plant tissue (Figs 2 and 5A) suggests that the CKs produced by *Magnaporthe* are also secreted and detected by the plant. The plant receptors and pathways engaged in CK detection remain to be identified and CK mutants in rice will have to be produced to address this question.

### Fungal cytokinins affect plant defense and nutrient fluxes and fungal physiology

Several observations support the hypothesis that the impaired virulence of *cks1* is the consequence of an inability of the fungus to manipulate the plant defense pathways and metabolic fluxes rather than a consequence of a self-triggered growth arrest (caused by the *cks1* loss-of-function) that would in turn trigger enhanced defense. First, enhanced defense is already visible *before* fungal penetration (Fig 5A and S5B Fig) at a time where the *cks1* and control strains were indistinguishable in terms of growth (S4C Fig). Second, reduced fungal growth could be partially restored by manipulating the plant in three ways: (i) by reducing plant defense chemically (Fig 4) (ii) by impairing immunity genetically in two independent mutants (Fig 6), (iii) by modifying plant fertilization (Fig 7A). Third, the restoration of virulence under high fertilization (Fig 7A) did not affect the over-induction of defense responses (Fig 7B). This indicates that the arrest of fungal growth can be compensated by high N-fertilization, although the associated over-induction of defense response cannot, presumably because CKs are still not produced. For these reasons, we propose that the impaired virulence of the *cks1* mutant is the consequence of the absence of CK production that would normally dampen defenses and modify nutrient fluxes for the pathogen’s benefit. Since the *cks1* mutant is also more susceptible to oxidative stress *in vitro* (S4 Fig), this may further reduce its global capacity to grow into plant tissues, especially if the plant ROS production is enhanced (Fig 4A). In that sense the *cks1* mutant is similar to the *des1* mutant which is required for dampening ROS-mediated plant defense [54].

### Possible effects of fungal CK on the plant metabolism

The transient and elevated sugar content in *cks1* compared to the *cks1* ^*CKS1*^ strain at 48 hpi (Fig 8A) is consistent with previous studies in other pathosystems that showed that an early and strong accumulation of soluble sugars, providing energy for the establishment of host defenses [63,79,80]. By contrast, the lower contents of glucose, fructose and sucrose found after 72 hpi with the *cks1* strain suggests that photosynthesis is reduced in plants infected by the CK-deficient strain. We propose that *Magnaporthe*-derived CKs contribute to prevention of photosynthesis breakdown during infection process, for instance by limiting oxidative stress generated through photorespiration and, in consequence, allowing the establishment of the biotrophic phase. Among the different amino acids quantified, the contents of two key amino acids (glutamate and aspartate), which are known to be essential for *M*. *oryzae* [62], were differently affected between mutant and control strains following infection (Fig 8A). In *Arabidopsis*, CKs were described to alter transcription of genes like glutamate dehydrogenase, asparagine synthetase and aspartate aminotransferase [81]. The fungal-derived CKs could also alter transcription of these genes to re-channel these amino acids towards fungal hyphae. Furthermore, CKs were previously shown to modify amino acid uptake through fungal cell membranes [82]; therefore, *cks1* mutants could also be affected in their capacity to import these molecules into fungal hyphae. Altogether, these possible effects of CKs could explain why aspartate and glutamate contents were differently affected during infection by *cks1* strain and the complemented *cks1* ^*CKS1*^ strain. These results suggest that fungal CKs could be involved in pathogen nutrition during infection as hypothesized for many plant/fungi interactions by Greene (1980) [16].

Our results also suggest that the lack of virulence of *cks1* mutants is partially due to an inability to limit plant defense responses like the oxidative burst (Fig 4) and transcription of defense-related genes (Fig 5A). The enhanced oxidative burst could lead to the stronger induction of defense markers observed during mutant infection and could participate to the strengthening of the cell wall to limit fungal penetration [83,84]. This is consistent with the ROS scavenging activity of CKs demonstrated in transgenic tobacco by Pogány et al., (2004)[85]. Quite paradoxically, exogenously applied CKs have been shown to enhance, in combination with salicylic acid, rice defense marker genes expression and phytoalexin biosynthesis [43,86]. This synergistic effect depends on key defense transcriptional regulators like OsWRKY45, a pivotal factor in biotic and abiotic stress responses [87]. Similarly, in *Arabidopsis*, specific recognition of bacterial CKs by plant CK receptors leads to a stronger induction of plant defenses and host resistance, involving plant CK Response Regulators and the transcription factor TGA3 [88]. In this context, how the CKs produced by *Magnaporthe oryzae* can act as negative regulators of defenses remains to be elucidated. A tight temporal and spatial production of CKs by the blast fungus could be central to avoiding enhanced activation of defenses.

### Concluding remarks

CKs play a key role in plant-microorganism communication [89] and it seems to be particularly true in plant symbiotic relations with fungi and bacteria [90,91]. Our work, showing that *Magnaporthe* requires CK production to be fully virulent, extends the key role that these hormones play in the interactions between plants and microbes to pathogenic fungi that do not trigger organ deformation. Our work suggests that CKs produced by *M*. *oryzae* act like classical effectors during host invasion as they significantly reduce defenses (e.g. [78]). Moreover, our study shows that fungal CKs can divert and attract plant nutrients essential for fungal growth, much like the TAL effectors from bacteria [3]. Therefore, CKs, like other hormones, could represent metabolic effectors with several biological functions. Given their central role in several metabolic processes, plant hormones represent ideal factors to be acquired as effectors by pathogens. Accordingly, evolution probably led the rice blast fungus to include CKs into its weapons for successful colonization of rice plants.

## Materials and Methods

### *In silico* analysis on *M*. *oryzae* and protein modelling

Based on published studies on plant CK metabolisms (cited in S1 Table), sequences from plant proteins were used to perform BLASTp on the *Magnaporthe* proteome at http://www.broadinstitute.org/annotation/genome/magnaporthe_grisea/Blast.html?sp=Sblastp. We used an E-value of 1e-3, with the comparison Matrix BLOSUM62 and gapped alignment. Similarly, BLASTp were performed on the yeast proteome available at http://www.yeastgenome.org/cgi-bin/blast-sgd.pl with default parameters. Hit proteins from yeast and *M*. *oryzae* were used to BLASTp back on *Magnaporthe* proteome to ensure Best Blast Mutual Hits were identified.

Protein structure predictions were realized with the on-line platform I-TASSER (http://zhanglab.ccmb.med.umich.edu/I-TASSER/). The primary sequences of proteins of interest were submitted (At2g27760 and MGG_04857) and secondary structures were predicted. Based on secondary structure predictions and primary sequences, the most similar proteins whose 3D structure was determined by NMR or X-ray crystallography were used as template for the model prediction. The model presented in S1B Fig was obtained with the MOD5 yeast protein (PDB accessions: 3epjA and 3ephA) as a template. Similar models were obtained with 3foz (*E*. *coli*) and 3a8t (*Humulus lupulus*) proteins as templates. Afterwards, alignments between the protein of interest and templates were generated by the use of different threading programs (MUSTER, FFAS-3D, SPARKS-X, HHSEARCH I, Neff-PPAS, HHSEARCH, pGenTHREADER, wdPPAS, PROSPECT2). Finally, the 10 best alignments were used to generate five structural models characterized [47]. The quality of structures used as templates were evaluated by Qmean server as well as the models obtained (http://swissmodel.expasy.org/qmean/cgi/index.cgi).

### Statistical analysis and experimental design

We analyzed results obtained for symptom quantification (Figs 1B, 3B and 4C) using a generalized linear model with a quasi-Poisson error structure. Significance was determined using a Chi² test. In each experiment, three biological replicates composed of 10 plants were analyzed per strain/condition. The size of the lesion trait was analyzed using a mixed model, with the “leaf” factor as random error, to compare the invasion of the different strains in the different conditions (Figs 1C and 3C). Boxplots represent data distribution using the median (indicated by the black line) and approximate quartiles. Each experiment was replicated at least three times. Gene expression data were analyzed using a Student t-test on four biological replicates, with each replicate composed of five to six plants (Figs 2, 5, 7B and S5). A student t-test was also used to analyze data presented in figs 1D, 6, 7A, 8, S3, S4, S6 and S7.

### Fungal transformation

Transformation was performed as described by Ribot et al., (2013)[92]. Protoplasts of GY11 were prepared as described previously [93]. For the knock-out *cks1* mutant, 1.2kb upstream and 1.2 kb downstream regions of the gene of interest were amplified by PCR using genomic GY11 DNA (100 ng) as template. The strategy used for constructing the gene replacement cassettes is derived from Kämper (2004) [94] and presented in the S2 Fig. Primers used are shown in S2 Table. Growing colonies were transferred to Tanaka-Hygromycin or Tanaka-Basta plates for assessing resistance. Resistant colonies were further grown on rice agar media for 7–10 days at 26°C, purified by single-spore isolation, tested for resistance, and stored at -20°C. At least 2 independent transgenic fungal lines were isolated for each plasmid. Resistant colonies were characterized by PCR using a Phire Plant Direct PCR kit (Thermo Scientific, Waltham, MA, USA).

### Measurement of cytokinin content

Fungal isolates were grown in 50mL of minimal Tanaka liquid medium without yeast extract (10g/L Glucose, 2 g/L NaNO_3_, 2 g/L KH_2_PO_4_, 0.5 g/L MgSO_4_-7H_2_O, 0.1 g/L CaCl_2_-2H_2_O, 4 mg/L FeSO_4_-7H_2_O, 1mg/L Thiamine, 5μg Biotin and microelements as Tanaka-B medium [95]) on rotary shaker for 10 days at 26°C. Yeast extract was excluded because this compound already contains hormones, including CKs, which leads to misinterpretation between CKs really produced compared to those that are just taken from the media and metabolized by the fungus. Fungal cultures were centrifuged for 10 min at 2000×g, the supernatant was collected and quantified. Mycelia were rinsed with sterile Tanaka liquid medium, centrifuged for 10 min at 2000×g and pressed with absorbent paper to accurately quantify the fungal biomass. Samples were then frozen and lyophilized. CK extraction and measurements were performed as previously described [19,50].

### Fungal and plant growth

Fungal isolates were grown on rice flour agar for spore production [96]. For the determination of interaction phenotypes and cytology analysis, a suspension of fungal conidiospores (5×10^4^ sp/mL) was spray-inoculated on the leaves of 3-week-old plants. For gene expression analysis, an inoculum of 2×10^5^ sp/mL was used. Radial mycelial growth was measured on minimal Tanaka solid medium (20g/L agar added to the recipe mentioned above), during 13 days at 26°C. Nipponbare plants (*O*. *sativa* ssp. *japonica*) were grown during three weeks as described previously [97]. In standard conditions, nitrogen fertilization was performed for three weeks and inoculation was done 4 days after fertilization. For the high/low nitrogen experiments, plants were fertilized for two weeks as in standard experiment, and in the third week, plants were fertilized (or not) one day before infection, as described in [61].

### Measurements of amino acids and sugar content

Amino acids and sugar contents were quantified as described in Gravot et al., (2010) [98] in leaf tissue of plants locally inoculated with a drop (15μL) of inoculum at 20 000 sp/mL. One centimeter corresponding to the inoculation site, and one centimeter above and below was sampled to quantify amino acids on 4 replicates composed of three leaf fragments (see S7 Fig for a picture of the experimental setting).

### RNA extraction and quantitative RT–PCR analysis

RNA extraction was performed as mentioned in Delteil et al, 2012 [57]. Quantitative PCR was performed using LC 480 SYBR Green I Master Mix (Roche, Basel, Switzerland) and a LightCycler 480 instrument (Roche). Amplification was performed as follows: 95°C for 10 min; 40 cycles of 95°C for 15 s, 60°C for 20 s and 72°C for 30 s; then 95°C for 5 min and 40°C for 30 s. In Figs 5 and 7, in order to compare genes with different expression levels, for a given gene, all values were normalized using the average value of this gene across the different conditions.

### Cytokinin and DPI treatments

Kinetin (Sigma) and cis-zeatin (OlChemIm) were diluted in 50% ethanol to prepare a stock solution of 50 mM. The solution sprayed contained 50 μM of CKs (replaced by ethanol 50% in mock solution), Tween at 0.02% final, diluted in water. Kinetin was applied to plants as described before [43] with slight modifications. Diphenylene iodonium (DPI; SIGMA D23926) treatment was performed at 0.5 μM final concentration, prepared from a 50× stock solution diluted in 50% DMSO. Mock treatment was performed with the equivalent volume of 50% DMSO/water. The volume sprayed was calculated to saturate the leaf surface (5mL for 10 three week-old plants).

### Tissue staining for confocal observation and H_2_O_2_ staining

Inoculated leaves were harvested, fixed and stained as described by Ballini et al., (2013) [61]. In order to show H_2_O_2_ accumulation, we performed a DAB staining as mentioned in Faivre-rampant et al., (2008) [97].

## Supporting Information

S1 Table Putative genes involved in CK metabolisms and signaling pathways, in *Oryza sativa*, *Arabidopsis thaliana*, *Magnaporthe oryzae* and *Saccharomyces cerevisiae*. CK biosynthesis in rice and *Arabidopsis* is well described. Based on homology between primary protein sequences some putative orthologous could be identified in *M*. *oryzae* and yeast by BLASTp. By the same way, primary sequences of plant enzymes already described to be involved in degradation of CKs were used to find orthologous in fungi. Finally, in plants, CK transduction signaling is performed by a multistep phosphorelay system, involving Histidine Kinase (HK) receptors, Histidine Phosphotransfer Proteins (HPt) and Response Regulators (only RR-A and RR-B are shown). Multistep phosphorelay is also already described in yeast and is conserved in *M*. *oryzae*. IPT: Isopentenyl Transferase; tRNA-IPT: tRNA-Isopentenyl Transferase; LOG: Lonely Guy; CYP735A: Cytochrome P450 monooxygenase; AK: Adenosine Kinase; CK-N-GT: Cytokinin N-Glucosyltransferase; ZOGT: Zeatin-O-glucosyltransferase; CKX: Cytokinin Oxydase. ^a^ BLASTp with AK from *A*. *thaliana*; ^b^ identified by BLASTp from yeast and plant enzymes; ^c^ found annotated in yeast genome and publications associated (www.yeastgenome.org)
(PDF)

S2 Table Primers used for RT-qPCR gene expression studies and for PCR genotyping. *MoCKS1*: *Magnaporthe oryzae Cytokinin Synthesis 1; OsRR1*, *2*, *3*, *6*, *10* corresponding to the annotation of response regulator coding genes published by Pareek et al., (2006) [53]. *PBZ1*: *Probenazole-inducible gene; CHI* and *CHI7* coding for chitinases; *PR*: *Pathogenesis Related genes*. The primers 1F, 2R, 1R, 3F and 3R are those used in S2 Fig
(PDF)

S1 Fig The MGG_04857 protein is similar to a tRNA-Isopentenyl transferase. (A) Primary sequence alignment of MOD5, AtIPT2 and the orthologous protein from *M*. *oryzae*. MOD5 and AtIPT2 are tRNA-isopentenyl transferases involved in CK biosynthesis in yeast [68] and *Arabidopsis* [24], respectively. Binding sites (underlined) are conserved in the putative tRNA-IPT from *M*. *oryzae* (MGG_04857, CKS1). The ATP binding site, from the amino acid 18 to 26 (GSTGTGKS), is conserved as well as tRNA binding sites (DAMQ 43–46 and T111) and DMAPP binding site (249–265). The percentage of identity and similarity between these proteins is higher than 30% and 50%, respectively (between MOD5 and AtIPT2: 32% identities, 55% positives (e-value 7.1^e^-27); MOD5 and putative tRNA-IPT from *M*. *oryzae*: 36% identities, 60% positives (8.6^e^-52); AtIPT2 and putative tRNA-IPT from *M*. *oryzae*: 36% identities, 54% positives (4^e^-40). (B) The putative tRNA-IPT from *Magnaporthe* has a predicted structure similar to the yeast MOD5 protein. MOD5 structure (light blue) was determined by X-ray diffraction [99]. The MOD5 structure used for the alignment corresponds to the 3ephA and 3epjA accessions in the Protein Data Bank (http://www.rcsb.org/pdb/home/home.do). The predicted structural model of tRNA-IPT from *M*. *oryzae* (MGG_04857; dark blue) was obtained by threading on the on-line platform I-TASSER, C-score 0,02 (Cf Materials and Methods). The quality scores of the structural model obtained for MGG_04857 and MOD5 by the Qmean server were 0.610 and 0.656 respectively. Conserved substrate binding sites are indicated by red arrows.
(PDF)

S2 Fig Characterization of fungal *cks1* mutants and complemented strains. (A) Genomic structure of *MoCKS1* (*MGG_04857*) gene. The *cks1* strain was generated by homologous recombination between the endogenous *CKS1* gene and a PCR fragment containing the hygromycin resistance gene. The knock-out mutation was complemented by a construct containing the genomic sequence of *MoCKS1* under its own promoter thus corresponding to the *cks1* ^*CKS1*^ control isolate (genomic sequence 1100nt upstream and 1646nt downstream the ATG). The position of primers used for genotyping and the length of PCR products are indicated. Genotyping was established by PCR (1.2% agarose gel). The genotype of the strains is indicated and * corresponds to negative controls. (B) PCR products were obtained with primers 1F-1R showed in (A) on both sides of the insertion site. (C) PCR products obtained with primers 1F-2R, demonstrate the presence of the hygromycin resistance gene replacing *CKS1* endogenous gene. (D) PCR products obtained with primers 3F-3R showing the presence of *MoCKS1* genomic sequence under its own promoter. (E) *In vitro* relative expression of *MoCKS1*, *MGG_04857*, in *cks1* strains (*cks1* n°3, *cks1* n°4), potential complemented strains *cks1* ^*CKS1*^ (*cks1* ^*CKS1*^ 4–1 and *cks1* ^*CKS1*^ 4–4; generated from *cks1* n°4 strain) and WT strain (GY11). The expression of *MoCKS1* was normalized with the expression of the *MG4* constitutive gene. The *cks1* n°3 and 4 strains do not express *MoCKS1*. We chose *cks1* n°4 as mutant strain and *cks1* ^*CKS1*^ 4–4 as control complemented strain. In all experiments presented, these strains were named *cks1* and *cks1* ^*CKS1*^. GY11 corresponds to the wild type genetic background used to generate fungal mutants.
(PDF)

S3 Fig Early developmental of *cks1* is not modified *in vitro* or on the plant surface. (A) The mycelial growth of the different strains was initiated from a fungal disc of 1cm of diameter from a first plate where the fungi reached maximum growth. The diameter of mycelia was measured during 13 days on minimal medium. The values are the mean and SD of 5 replicates per strain. (B) The development of the appressorium was measured on glass slides at the indicated time points for each strain and (C) on the plant leaf surface. Plants were inoculated and the frequency of spores showing complete appressorial development was measured. There was no significant difference between *cks1* (black bars) and *cks1* ^*CKS1*^ (white bars) strains as estimated with a t-test.
(PDF)

S4 Fig The *cks1* mutant is less tolerant to oxidative stress. The *cks1* mutant and complemented strain were grown on minimal medium containing H_2_O_2_ and radial growth was measured as a read-out of fungal fitness. (A) The *cks1* mutant is hypersensitive to oxidative stress. (*: P<0.001; t-test comparing *cks1* and complemented mutant strains of 5 replicates). (B) The effect of 1 mM H_2_O_2_ was tested in the presence of 25μM of kinetin.
(PDF)

S5 Fig The *cks1* mutant triggers different host CK signaling and defense transcriptional responses during early stages of infection. (A) The transcriptional regulation of CK marker genes (*OsRR2* (Os12g04500), *OsRR3* (Os01g72330) and *OsRR10* (Os02g35180) as named by Pareek et al., (2006) [53] was evaluated by quantitative RT-PCR using the *Actin* gene for normalization. (B) The expression of defense marker genes was also measured: *Os01g03390 (BB trypsin inhibitor)*, *Os11g37970 (HEL protein)*, *Os07g48020 (Peroxidase)*. Nipponbare plants were inoculated with spore suspension (in gelatin 0.5%) of either the *cks1* mutant (black bars) or *cks1* ^*CKS1*^ control strain (white bars) and gene expression was measured at 2, 4 and 6 hours post inoculation (hpi), before penetration of the leaf tissues. The values presented are the Log2 ratios (infected/not infected) of the means calculated from four independent replicates. Uninfected plants were sprayed with gelatin 0.5% but without spore suspension. This experiment was repeated twice and showed similar results. A t-test was used to compare the means of expression quantified in *cks1* (black bars) and *cks1* ^*CKS1*^ (white bars) inoculated plants. *: p-value < 0.05; **: p-value < 0.03; ***: p-value < 0.001.
(PDF)

S6 Fig The reduced virulence of *cks1* strain is restored by exogenous application of cis-zeatin. Plants were treated with 50μM of cis-zeatin or buffer alone 24h after inoculation with *cks1* mutant and *cks1* ^*CKS1*^ complemented strain. The symptoms were observed 6dpi (A) and the number of lesion per leaf is shown (B). The values represent the mean and SD of three biological replicates of 10 individuals. The different letters indicate significant differences between values obtained by t-test (p-value<0.04).
(PDF)

S7 Fig Accumulation of amino acids at the infected site and around. Sugar and amino acid contents were quantified (presented in nmol/mg of fresh weight), during infection (times are indicated), at the site of inoculation corresponding to the”infected zone” and one centimeter apart (respectively named “lower” and “upper non-infected zones”). For more details see Materials and Methods. A T-test (*, p-value < 0.05) was used to compare amino acid contents in leaf fragment from plants inoculated with the *cks1* (black bars) and *cks1* ^*CKS1*^ (white bars).
(PDF)

S8 Fig The tRNA-IPT protein is highly conserved across Ascomycetes. Phylogenetic tree based on the primary sequence of putative orthologous tRNA-IPT proteins identified by BLASTp in Ascomycetes. Multiple protein sequences alignment was performed with MUSCLE and alignment curation with Gblocks. The phylogenetic tree was obtained using PhyML [100]. The phylogenetic tree was rooted with the farthest species, *Pyronema omphalodes*. The different classes of Ascomycetes and bootstrap values are indicated.
(PDF)
