# Supplementary material for: Cytokinin Production by the Rice Blast Fungus Is a Pivotal Requirement for Full Virulence
Source: PLoS Pathog. 2016 Feb 22;12(2):e1005457. doi: 10.1371/journal.ppat.1005457 (PMC4765853; doi:10.1371/journal.ppat.1005457)
Supplement: S1 Table — CK biosynthesis in rice and Arabidopsis is well described. Based on homology between primary protein sequences some putative orthologous could be identified in M. oryzae and yeast by BLASTp. By the same way, primary sequences of plant enzymes already described to be involved in degradation of CKs were used to find orthologous in fungi. Finally, in plants, CK transduction signaling is performed by a multistep phosphorelay system, involving Histidine Kinase (HK) receptors, Histidine Phosphotransfer Proteins (HPt) and Response Regulators (only RR-A and RR-B are shown). Multistep phosphorelay is also already described in yeast and is conserved in M. oryzae. IPT: Isopentenyl Transferase; tRNA-IPT: tRNA-Isopentenyl Transferase; LOG: Lonely Guy; CYP735A: Cytochrome P450 monooxygenase; AK: Adenosine Kinase; CK-N-GT: Cytokinin N-Glucosyltransferase; ZOGT: Zeatin-O-glucosyltransferase; CKX: Cytokinin Oxydase. a BLASTp with AK from A. thaliana; b identified by BLASTp from yeast and plant enzymes; c found annotated in yeast genome and publications associated (www.yeastgenome.org) (PDF) [file ppat.1005457.s001.pdf]

Function	Family	Number of genes in *A.thaliana*	Number of genes in *O.sativa*	Number of genes in *M.oryzae*	Number of gene in *S.cerevisiae*
**Biosynthesis**	IPT	7[1,2,3]	8[11,12]	not found	not found
	tRNA-IPT	2 [4,5]	2[11]	1[b]	1[c]
	LOG	9[6]	11[11]	1[b]	1[c]
	CYP735A	2[7]	2[11]	difficult to identify	2[c]
	AK	2[8] (3 TAIR)	4[a]	1[b]	1[c]
**Degradation**	CKX	7[9]	11[11]	a lot of enzymes with a putative oxidase activity were found but their role in CKs metabolism is still unclear in fungi	
**Transduction signaling**	HKs and related proteins	16[10]	14[13]	10[14, 15, 16]	1[18,c]
	HPts	6[10]	5[13]	1[b]	1[18, 19c]
	RR-A	11[10]	22[13]; 10[11]	1[17, b]	1[18, 20,c]
	RR-B	12[10]	7[13]; 13[11]	1[17, b]	1[20,c]

[1]Kakimoto, T. (2001) Identification of plant cytokinin biosynthetic enzymes as dimethylallyl diphosphate:ATP/ADP isopentenyltransferases. *Plant Cell Physiol.* 42, 677–685; [2]Kakimoto, T. (2003) Biosynthesis of cytokinins. *J. Plant Res.* 116, 233–239; [3]Takei, K. *et al.* (2001) Identification of Genes Encoding Adenylate Isopentenyltransferase, a Cytokinin Biosynthesis Enzyme, in Arabidopsis thaliana. *J. Biol. Chem.* 276, 26405–26410; [4]Golovko, A. *et al.* (2002) Identification of a tRNA isopentenyltransferase gene from Arabidopsis thaliana. *Plant Mol. Biol.* 49, 161–169; [5]Miyawaki, K. *et al.* (2006) Roles of Arabidopsis ATP/ADP isopentenyltransferases and tRNA isopentenyltransferases in cytokinin biosynthesis. *Proc. Natl. Acad. Sci.*; [6]Kuroha, T. *et al.* (2009) Functional analyses of LONELY GUY cytokinin-activating enzymes reveal the importance of the direct activation pathway in Arabidopsis. *Plant Cell* 21, 3152–3169.; [7]Takei, K. *et al.* (2004) Arabidopsis CYP735A1 and CYP735A2 encode cytokinin hydroxylases that catalyze the biosynthesis of trans-Zeatin. *J. Biol. Chem.* 279, 41866–41872; [8]Moffatt, B. a *et al.* (2000) Adenosine kinase of Arabidopsis. Kinetic properties and gene expression. *Plant Physiol.* 124, 1775–1785; [9]Schmülling, T. *et al.* (2003) Structure and function of cytokinin oxidase/dehydrogenase genes of maize, rice, Arabidopsis and other species. *J. Plant Res.* 116, 241–52; [10]Hwang, I. and Sheen, J. (2001) Two-component circuitry in Arabidopsis cytokinin signal transduction. *Nature* 413, 383–9; [11]Tsai, Y.-C. *et al.* (2012) Characterization of genes involved in cytokinin signaling and metabolism from rice. *Plant Physiol.* 158, 1666–84; [12]Sakamoto, T. *et al.* (2006) Ectopic expression of KNOTTED1-like homeobox protein induces expression of cytokinin biosynthesis genes in rice. *Plant Physiol.* 142, 54–62; [13]Pareek, A. *et al.* (2006) Whole-genome analysis of Oryza sativa reveals similar architecture of two-component signaling machinery with Arabidopsis. *Plant Physiol.* 142, 380–97; [14]Dean, R. a *et al.* (2005) The genome sequence of the rice blast fungus Magnaporthe grisea. *Nature* 434, 980–986; [15]Motoyama, T. *et al.* (2005) A two-component histidine kinase of the rice blast fungus is involved in osmotic stress response and fungicide action. *Fungal Genet. Biol.* 42, 200–212; [16]Zhang, H. *et al.* (2010) A two-component histidine kinase, MoSLN1, is required for cell wall integrity and pathogenicity of the rice blast fungus, Magnaporthe oryzae. *Curr. Genet.* 56, 517–528; [17]Motoyama, T. *et al.* (2008) Involvement of putative response regulator genes of the rice blast fungus Magnaporthe oryzae in osmotic stress response, fungicide action, and pathogenicity. *Current Genetics.* 54, 185-195; [18]Posas, F. *et al.* (1996) Yeast HOG1 MAP kinase cascade is regulated by a multistep phosphorelay mechanism in the SLN1-YPD1-SSK1 “two-component” osmosensor. *Cell* 86, 865–875; [19]Xu, Q. and West, a H. (1999) Conservation of structure and function among histidine-containing phosphotransfer (HPt) domains as revealed by the crystal structure of YPD1. *J. Mol. Biol.* 292, 1039–1050; [20]Li, S. *et al.* (1998) The yeast histidine protein kinase, Sln1p, mediates phosphotransfer to two response regulators, Ssk1p and Skn7p. *EMBO J.* 17, 6952–6962
