# Supplementary material for: Cytokinin Production by the Rice Blast Fungus Is a Pivotal Requirement for Full Virulence
Source: PLoS Pathog. 2016 Feb 22;12(2):e1005457. doi: 10.1371/journal.ppat.1005457 (PMC4765853; doi:10.1371/journal.ppat.1005457)
Supplement: S2 Table — MoCKS1: Magnaporthe oryzae Cytokinin Synthesis 1; OsRR1, 2, 3, 6, 10 corresponding to the annotation of response regulator coding genes published by Pareek et al., (2006) [53]. PBZ1: Probenazole-inducible gene; CHI and CHI7 coding for chitinases; PR: Pathogenesis Related genes. The primers 1F, 2R, 1R, 3F and 3R are those used in S2 Fig (PDF) [file ppat.1005457.s002.pdf]

Suppl Table 2

Primer name	Accession	Annotation	Sequence
	MGG_04857	MoCKS1	F-GCAACAAGACGTGCGAAGTA
			R-TTATGGCGGTGACCTCTGAT
	Os11g04720	OsRR1	F-CTCAAACCAGTTCGGCTCTC
			R-CTGTTGTTGCTTTCGCTGTT
	Os12g04500	OsRR2	F-CTCAAACCAGTTCGGCTCTC
			R-TGTTGCTTTGCAGGTGTTGT
	Os01g72330	OsRR3	F-ATCTGCTCAAGAGGGTGAAGGG
			R-TGTCAGCGAGCTTGACAGGTTTC
	Os04g57720	OsRR6	F-TGTCAGGCATTTGCTTGTTC
			R-GGTGCCATTTCAACATTGTG
	Os02g35180	OsRR10	F-TCCAGAAGGCTGGATTGATT
			R-GCTGCACTCTTGCTTGATGA
	Os12g36880	PBZ1	F-AGGCATCAGTGGTCAGTAGAG
			R-CGGGTCTTGTATGTGCTTCC
	Os07g35560	CHI	F-TTAACGGCGCTGCTACCATT
			R-TCCCATCCTCTTACTGCCGA
	Os01g03390	BB trypsin inhibitor	F-ATCTGTGTCCGTCAATAAAACTCG
			R-TTGCTCTTGGTCACTGGCTAG
	Os07g48020	POX	F-GGATGCGTTCGTTGCTGGAAG
			R-GCTGCTCTGCTCCATACACTTG
	Os11g37970	HEL protein	F-AGTATGGATGGACCGCCTTCTG
			R-CGCAATTATTGTCGCACCTGTTC
	Os06g51050	CHI7	F-CAATGCACACGAGATTGTGA
			R-CCGCATTGTGTTAACGTCCA
	Os12g36850	PR10	F-CAGATGATCGAGGCGTACCT
			R-CCACGCCACAGTAACATGAC
	Os12g43430	PR5	F-AGCCAGGACTTCTACGACCT
			R-GCGTGTGTCTTGGTGTTGTC
1F	MGG_04857	promotor MGG_04857	GAGATGATGGGCGTGATACC
2R		hygromycin resistance gene	GGGATCAGCAATCGCGCATATGA
1R	MGG_04857	terminator MGG_04857	TTTGCTAGGCTTCGGTGAAT
3F	MGG_04857	Not I_ promotor MGG_04857	atatgcggccgcCCACATGATGGACTCGCAGAT
3R	MGG_04857	EcoRI_ 2nd exon MGG_04857	atatgaattcTTACTGGTTGTCCTCAAAACGGATAG
