# Supplementary figures and images for: Cytokinin Production by the Rice Blast Fungus Is a Pivotal Requirement for Full Virulence
Source: PLoS Pathog. 2016 Feb 22;12(2):e1005457. doi: 10.1371/journal.ppat.1005457 (PMC4765853; doi:10.1371/journal.ppat.1005457)

Suppl Figure 1

A.

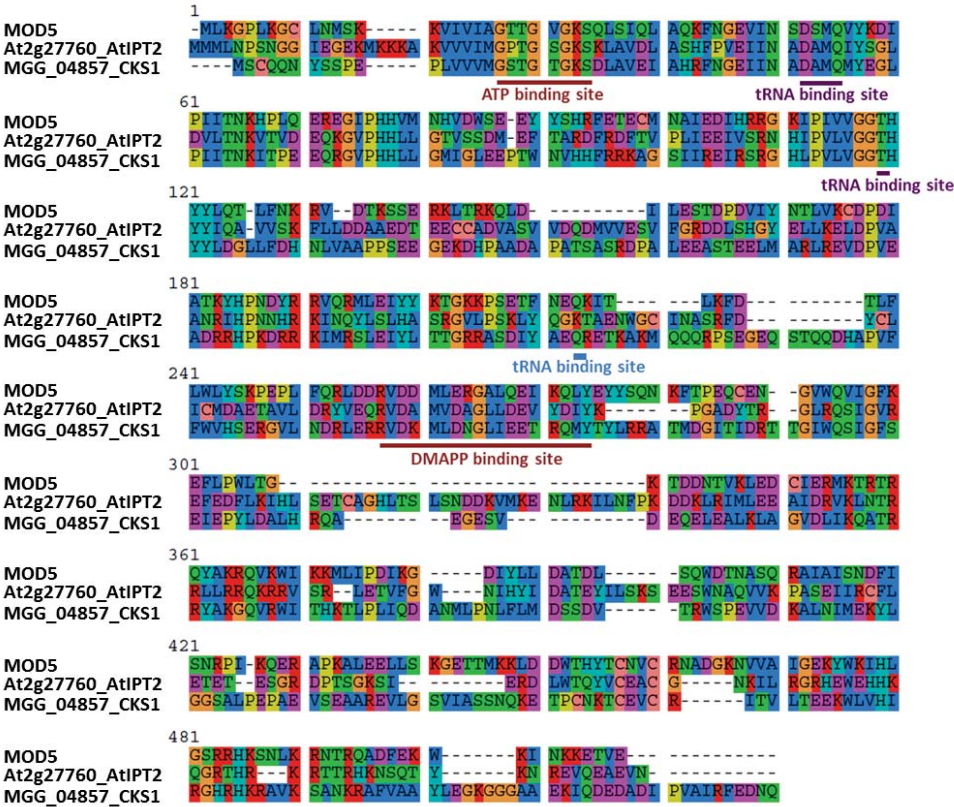

B.

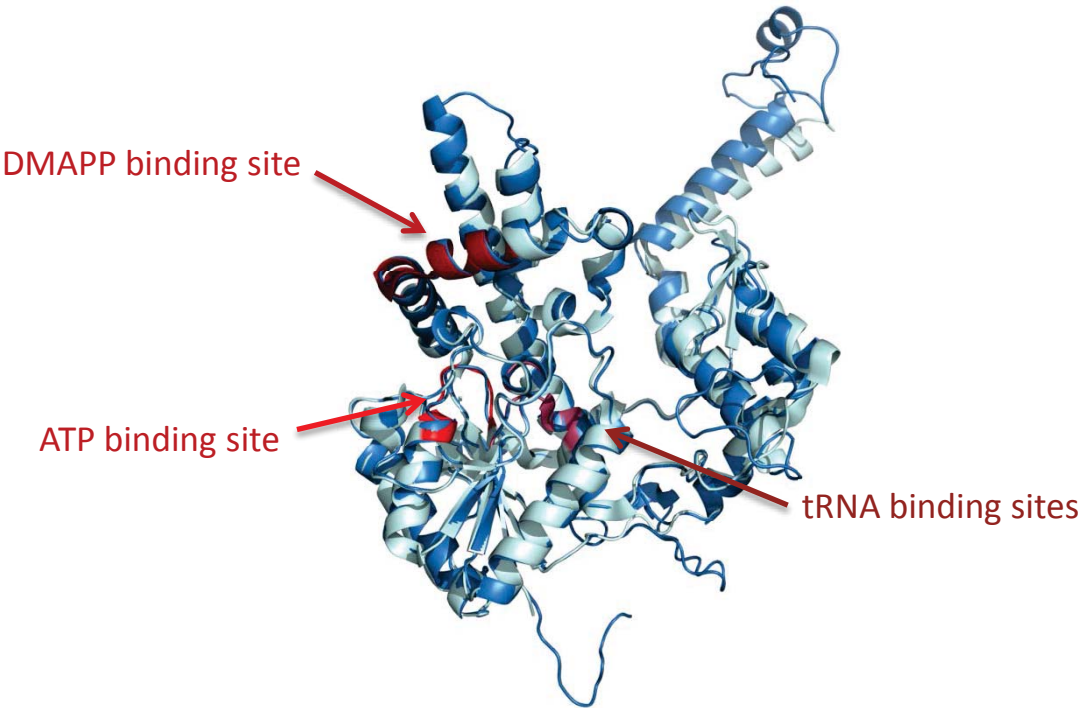

Supplement: S1 Fig — (A) Primary sequence alignment of MOD5, AtIPT2 and the orthologous protein from M. oryzae. MOD5 and AtIPT2 are tRNA-isopentenyl transferases involved in CK biosynthesis in yeast [68] and Arabidopsis [24], respectively. Binding sites (underlined) are conserved in the putative tRNA-IPT from M. oryzae (MGG_04857, CKS1). The ATP binding site, from the amino acid 18 to 26 (GSTGTGKS), is conserved as well as tRNA binding sites (DAMQ 43–46 and T111) and DMAPP binding site (249–265). The percentage of identity and similarity between these proteins is higher than 30% and 50%, respectively (between MOD5 and AtIPT2: 32% identities, 55% positives (e-value 7.1e-27); MOD5 and putative tRNA-IPT from M. oryzae: 36% identities, 60% positives (8.6e-52); AtIPT2 and putative tRNA-IPT from M. oryzae: 36% identities, 54% positives (4e-40). (B) The putative tRNA-IPT from Magnaporthe has a predicted structure similar to the yeast MOD5 protein. MOD5 structure (light blue) was determined by X-ray diffraction [99]. The MOD5 structure used for the alignment corresponds to the 3ephA and 3epjA accessions in the Protein Data Bank (http://www.rcsb.org/pdb/home/home.do). The predicted structural model of tRNA-IPT from M. oryzae (MGG_04857; dark blue) was obtained by threading on the on-line platform I-TASSER, C-score 0,02 (Cf Materials and Methods). The quality scores of the structural model obtained for MGG_04857 and MOD5 by the Qmean server were 0.610 and 0.656 respectively. Conserved substrate binding sites are indicated by red arrows. (PDF) [file ppat.1005457.s003.pdf]

## Suppl Figure 2

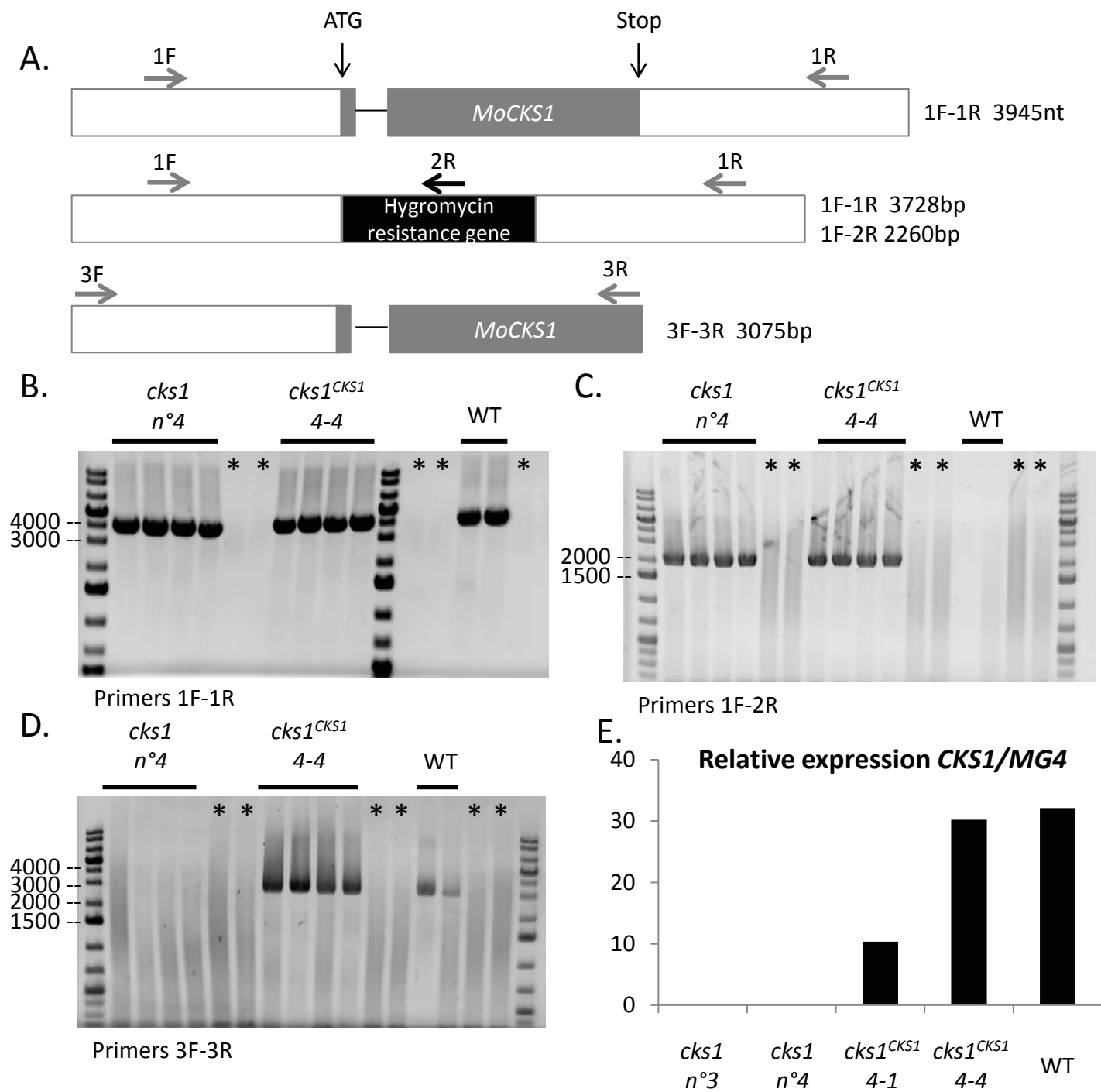

Supplement: S2 Fig — (A) Genomic structure of MoCKS1 (MGG_04857) gene. The cks1 strain was generated by homologous recombination between the endogenous CKS1 gene and a PCR fragment containing the hygromycin resistance gene. The knock-out mutation was complemented by a construct containing the genomic sequence of MoCKS1 under its own promoter thus corresponding to the cks1 CKS1 control isolate (genomic sequence 1100nt upstream and 1646nt downstream the ATG). The position of primers used for genotyping and the length of PCR products are indicated. Genotyping was established by PCR (1.2% agarose gel). The genotype of the strains is indicated and * corresponds to negative controls. (B) PCR products were obtained with primers 1F-1R showed in (A) on both sides of the insertion site. (C) PCR products obtained with primers 1F-2R, demonstrate the presence of the hygromycin resistance gene replacing CKS1 endogenous gene. (D) PCR products obtained with primers 3F-3R showing the presence of MoCKS1 genomic sequence under its own promoter. (E) In vitro relative expression of MoCKS1, MGG_04857, in cks1 strains (cks1 n°3, cks1 n°4), potential complemented strains cks1 CKS1 (cks1 CKS1 4–1 and cks1 CKS1 4–4; generated from cks1 n°4 strain) and WT strain (GY11). The expression of MoCKS1 was normalized with the expression of the MG4 constitutive gene. The cks1 n°3 and 4 strains do not express MoCKS1. We chose cks1 n°4 as mutant strain and cks1 CKS1 4–4 as control complemented strain. In all experiments presented, these strains were named cks1 and cks1 CKS1. GY11 corresponds to the wild type genetic background used to generate fungal mutants. (PDF) [file ppat.1005457.s004.pdf]

### Suppl Figure 3

A.

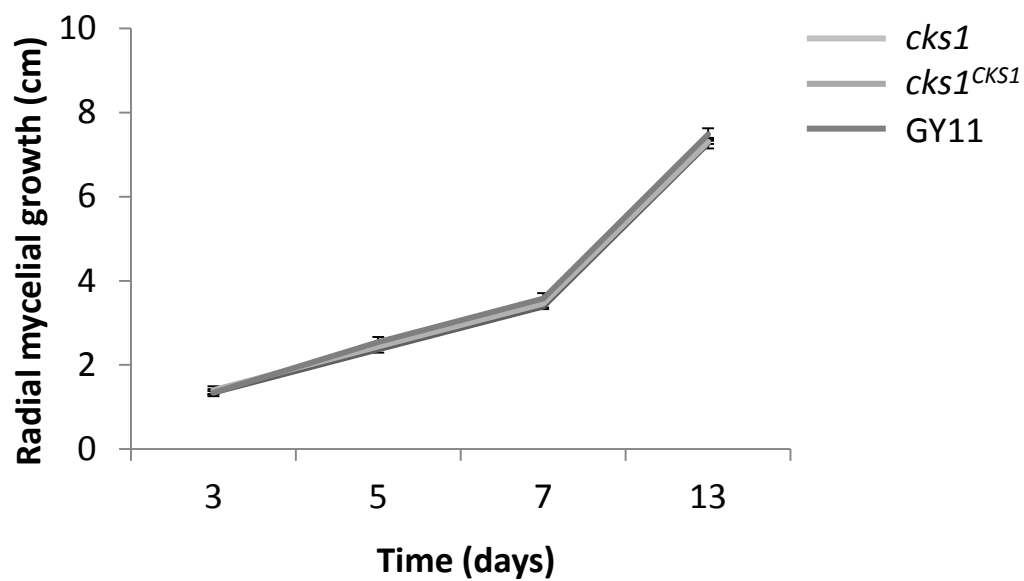

B.

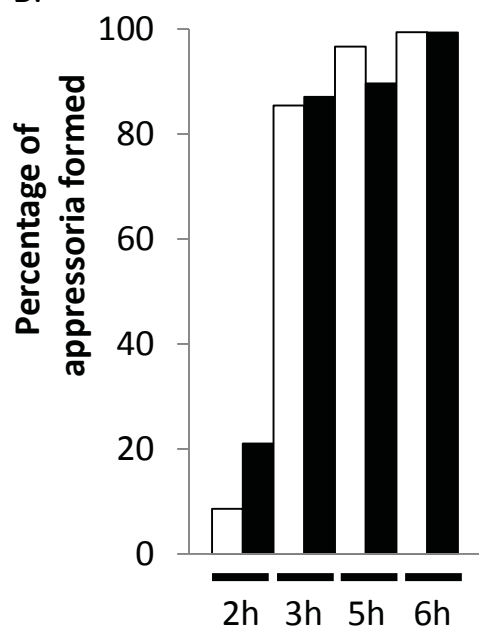

C.

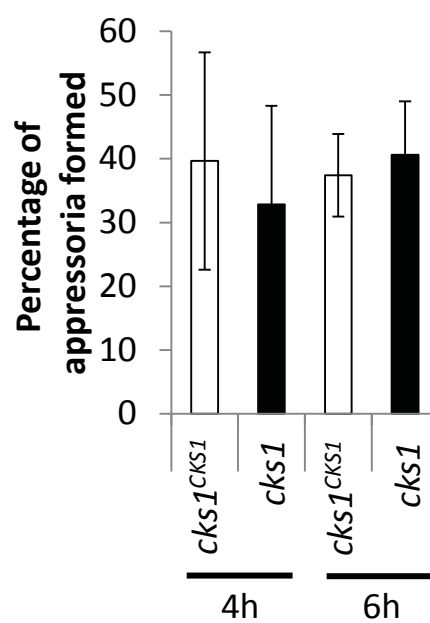

Supplement: S3 Fig — (A) The mycelial growth of the different strains was initiated from a fungal disc of 1cm of diameter from a first plate where the fungi reached maximum growth. The diameter of mycelia was measured during 13 days on minimal medium. The values are the mean and SD of 5 replicates per strain. (B) The development of the appressorium was measured on glass slides at the indicated time points for each strain and (C) on the plant leaf surface. Plants were inoculated and the frequency of spores showing complete appressorial development was measured. There was no significant difference between cks1 (black bars) and cks1 CKS1 (white bars) strains as estimated with a t-test. (PDF) [file ppat.1005457.s005.pdf]

Suppl Figure 4

A.

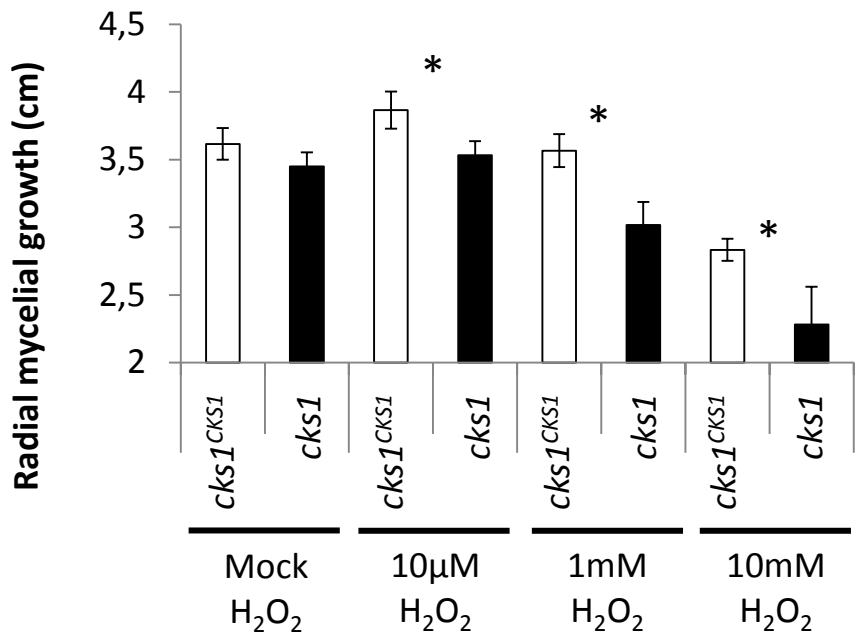

B.

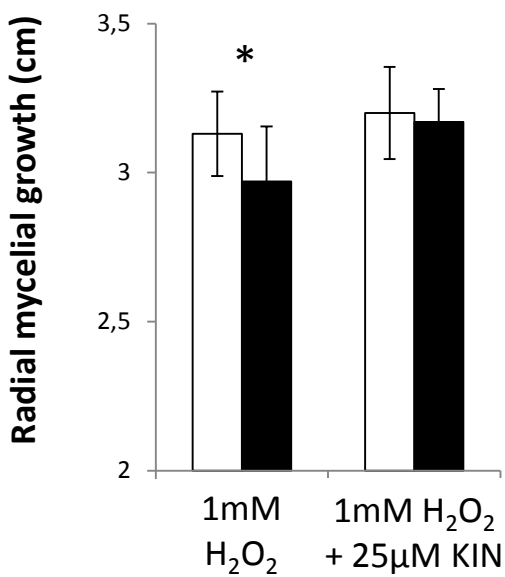

Supplement: S4 Fig — The cks1 mutant and complemented strain were grown on minimal medium containing H2O2 and radial growth was measured as a read-out of fungal fitness. (A) The cks1 mutant is hypersensitive to oxidative stress. (*: P<0.001; t-test comparing cks1 and complemented mutant strains of 5 replicates). (B) The effect of 1 mM H2O2 was tested in the presence of 25μM of kinetin. (PDF) [file ppat.1005457.s006.pdf]

Suppl Figure 5

A.

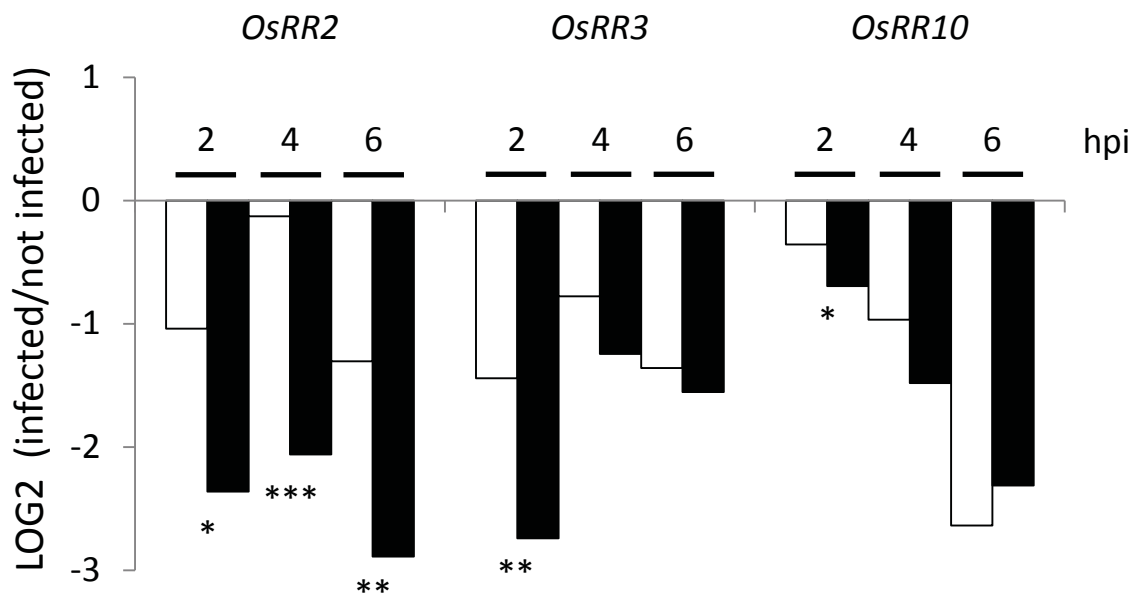

B.

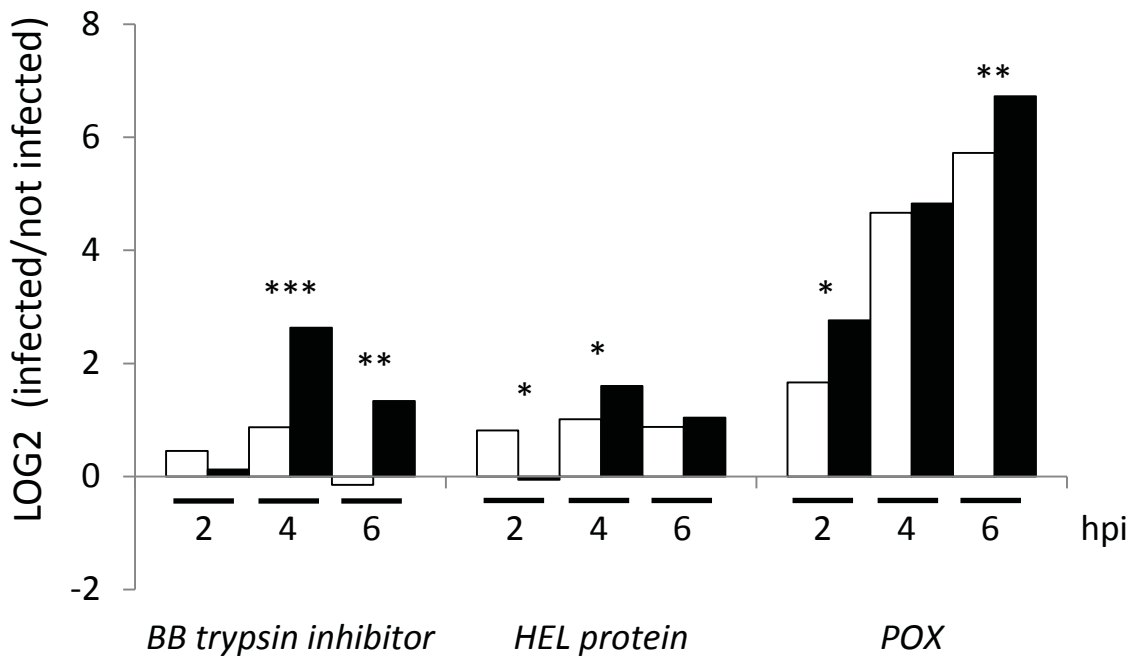

Supplement: S5 Fig — (A) The transcriptional regulation of CK marker genes (OsRR2 (Os12g04500), OsRR3 (Os01g72330) and OsRR10 (Os02g35180) as named by Pareek et al., (2006) [53] was evaluated by quantitative RT-PCR using the Actin gene for normalization. (B) The expression of defense marker genes was also measured: Os01g03390 (BB trypsin inhibitor), Os11g37970 (HEL protein), Os07g48020 (Peroxidase). Nipponbare plants were inoculated with spore suspension (in gelatin 0.5%) of either the cks1 mutant (black bars) or cks1 CKS1 control strain (white bars) and gene expression was measured at 2, 4 and 6 hours post inoculation (hpi), before penetration of the leaf tissues. The values presented are the Log2 ratios (infected/not infected) of the means calculated from four independent replicates. Uninfected plants were sprayed with gelatin 0.5% but without spore suspension. This experiment was repeated twice and showed similar results. A t-test was used to compare the means of expression quantified in cks1 (black bars) and cks1 CKS1 (white bars) inoculated plants. *: p-value < 0.05; **: p-value < 0.03; ***: p-value < 0.001. (PDF) [file ppat.1005457.s007.pdf]

Suppl Figure 6

A.

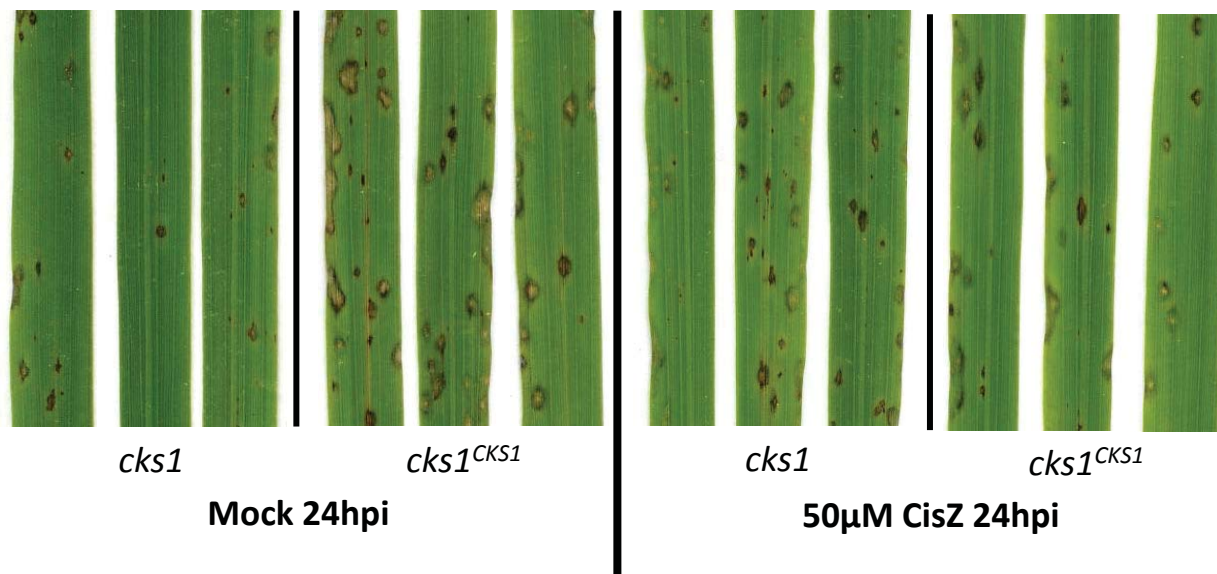

B.

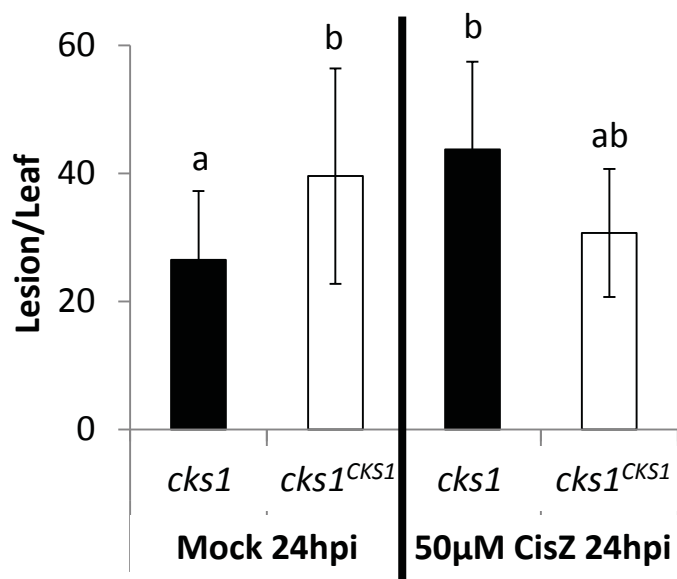

Supplement: S6 Fig — Plants were treated with 50μM of cis-zeatin or buffer alone 24h after inoculation with cks1 mutant and cks1 CKS1 complemented strain. The symptoms were observed 6dpi (A) and the number of lesion per leaf is shown (B). The values represent the mean and SD of three biological replicates of 10 individuals. The different letters indicate significant differences between values obtained by t-test (p-value<0.04). (PDF) [file ppat.1005457.s008.pdf]

Supl Figure 8

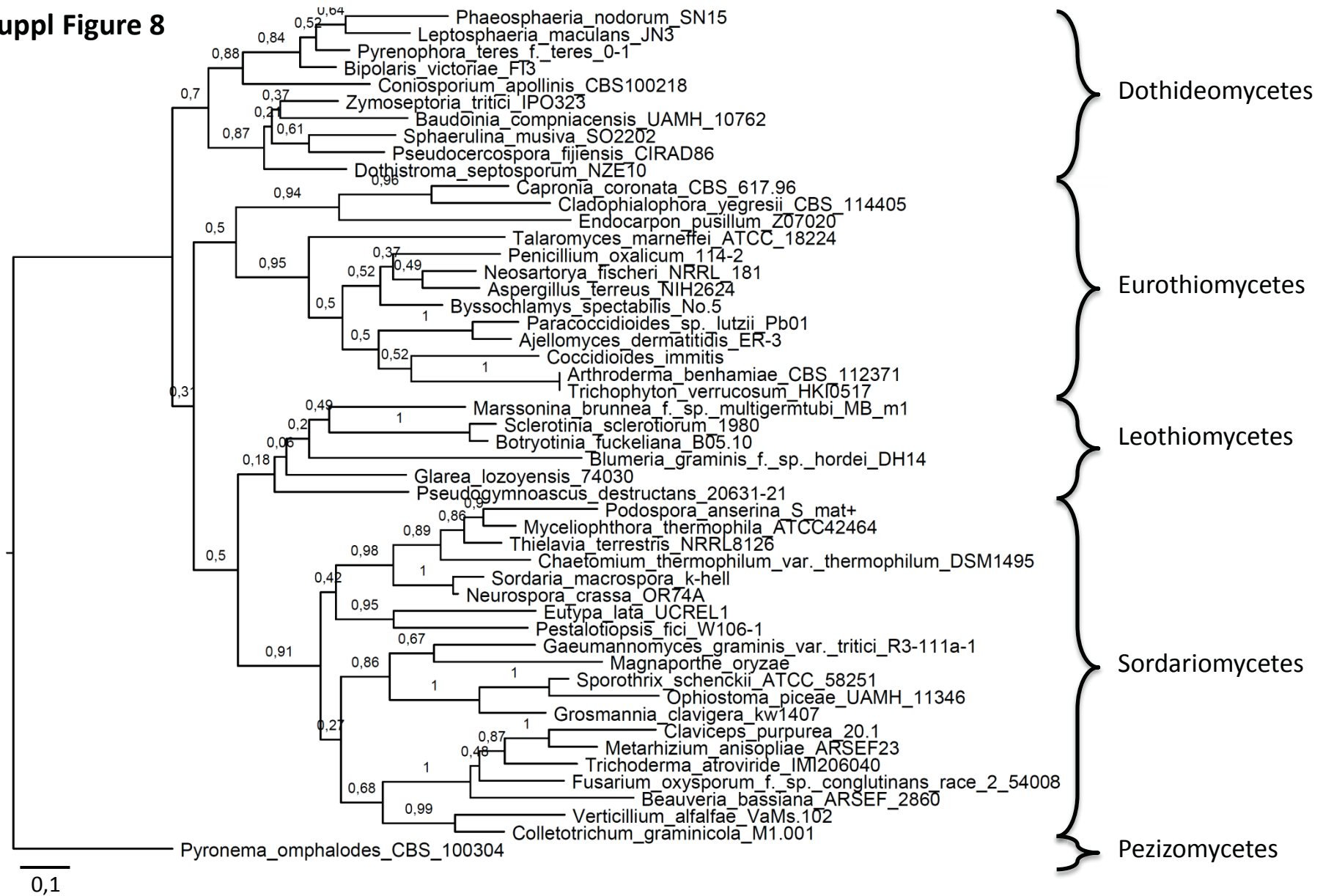

Supplement: S8 Fig — Phylogenetic tree based on the primary sequence of putative orthologous tRNA-IPT proteins identified by BLASTp in Ascomycetes. Multiple protein sequences alignment was performed with MUSCLE and alignment curation with Gblocks. The phylogenetic tree was obtained using PhyML [100]. The phylogenetic tree was rooted with the farthest species, Pyronema omphalodes. The different classes of Ascomycetes and bootstrap values are indicated. (PDF) [file ppat.1005457.s010.pdf]
