# Supplementary material for: Cytokinin Production by the Rice Blast Fungus Is a Pivotal Requirement for Full Virulence
Source: PLoS Pathog. 2016 Feb 22;12(2):e1005457. doi: 10.1371/journal.ppat.1005457 (PMC4765853; doi:10.1371/journal.ppat.1005457)
Supplement: S7 Fig — Sugar and amino acid contents were quantified (presented in nmol/mg of fresh weight), during infection (times are indicated), at the site of inoculation corresponding to the”infected zone” and one centimeter apart (respectively named “lower” and “upper non-infected zones”). For more details see Materials and Methods. A T-test (*, p-value < 0.05) was used to compare amino acid contents in leaf fragment from plants inoculated with the cks1 (black bars) and cks1 CKS1 (white bars). (PDF) [file ppat.1005457.s009.pdf]

Suppl Figure 7

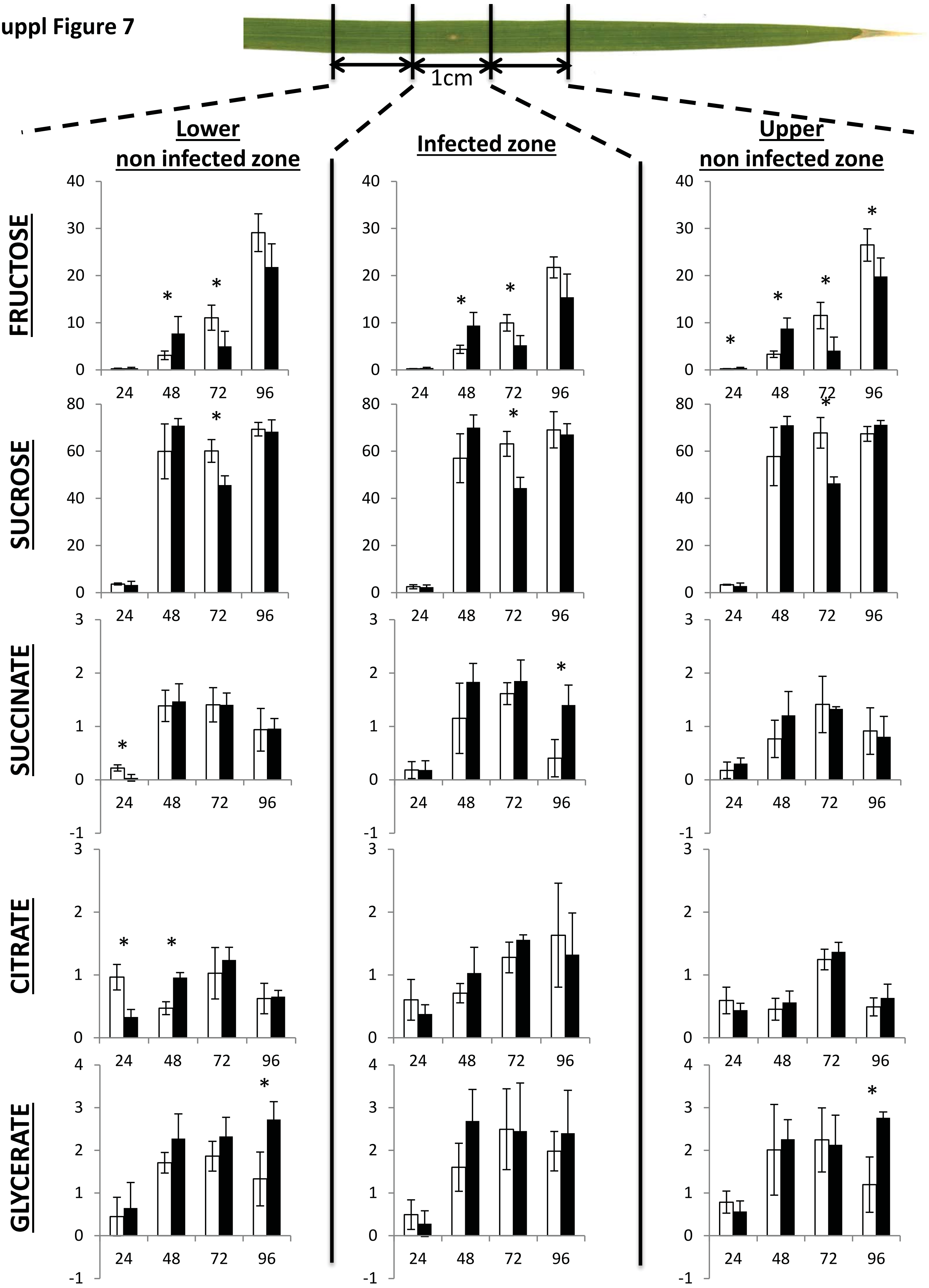

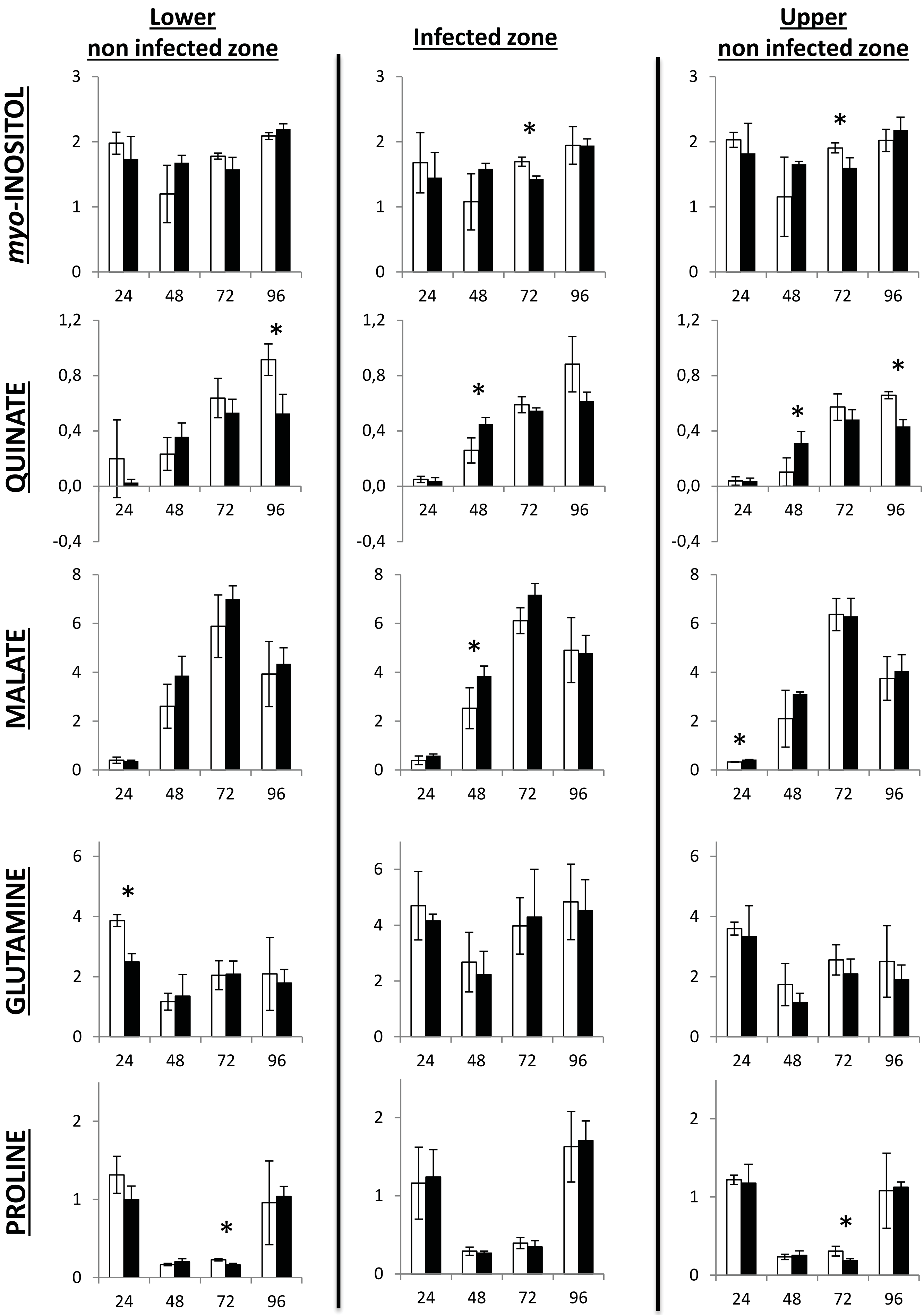

Lower
non infected zone
Infected zone
Upper
non infected zone
myo-INOSITOL
QUINATE
MALATE
GLUTAMINE
PROLINE
24
48
72
96
*

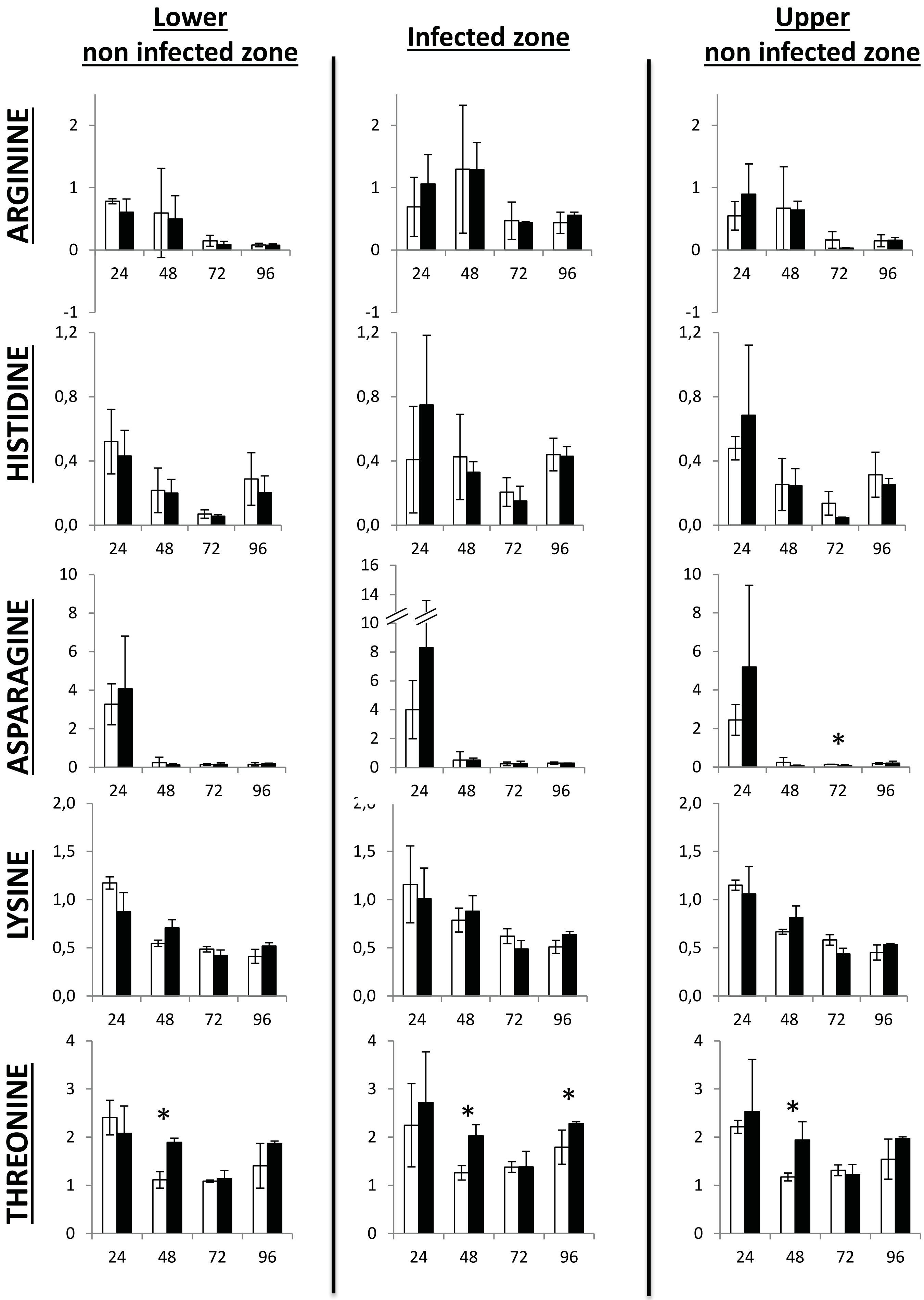

Lower non infected zone
Infected zone
Upper non infected zone
ARGININE
HISTIDINE
ASPARAGINE
LYSINE
THREONINE
24
48
72
96
*

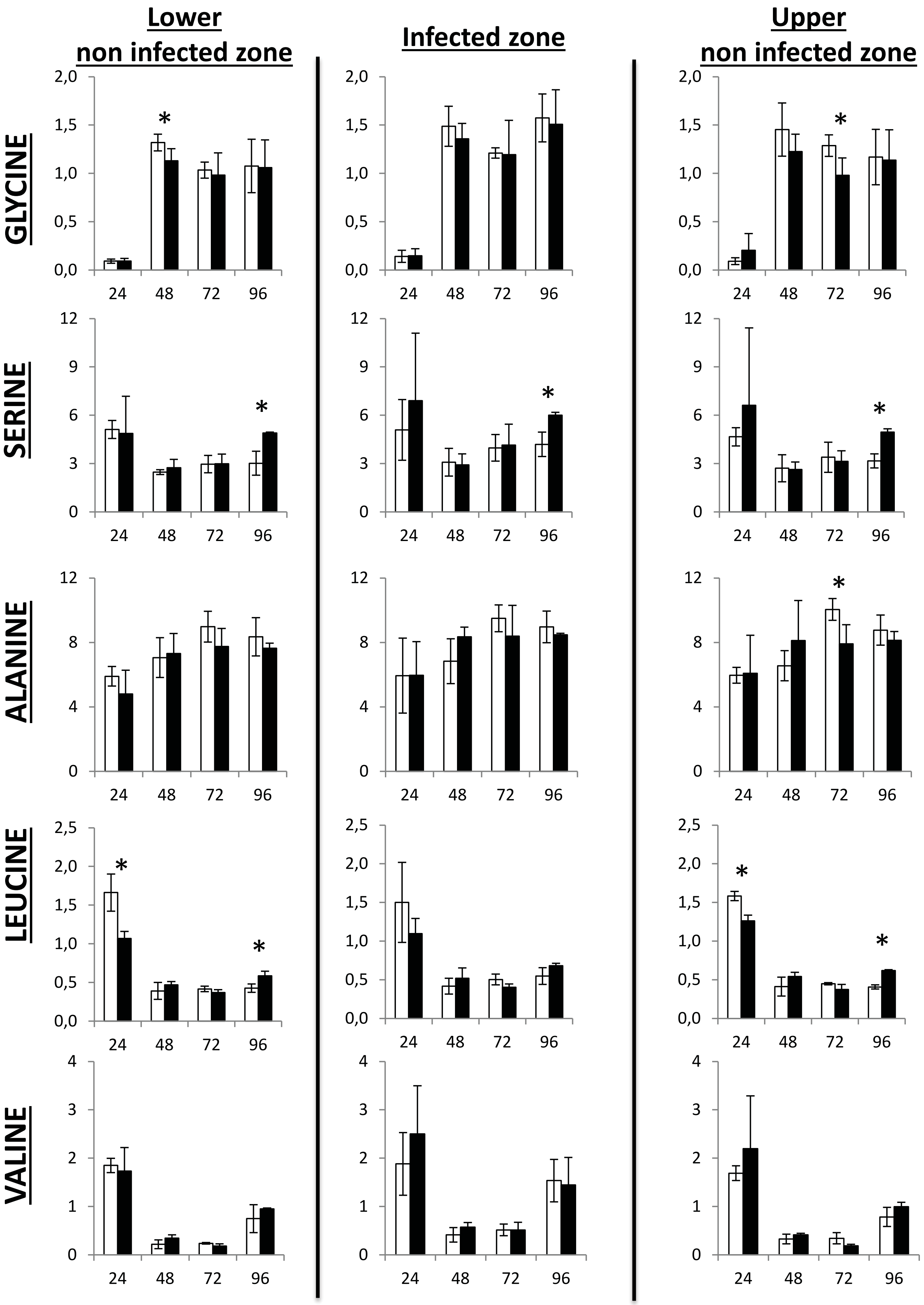

Lower non infected zone
Infected zone
Upper non infected zone
GLYCINE
SERINE
ALANINE
LEUCINE
VALINE
24
48
72
96
*

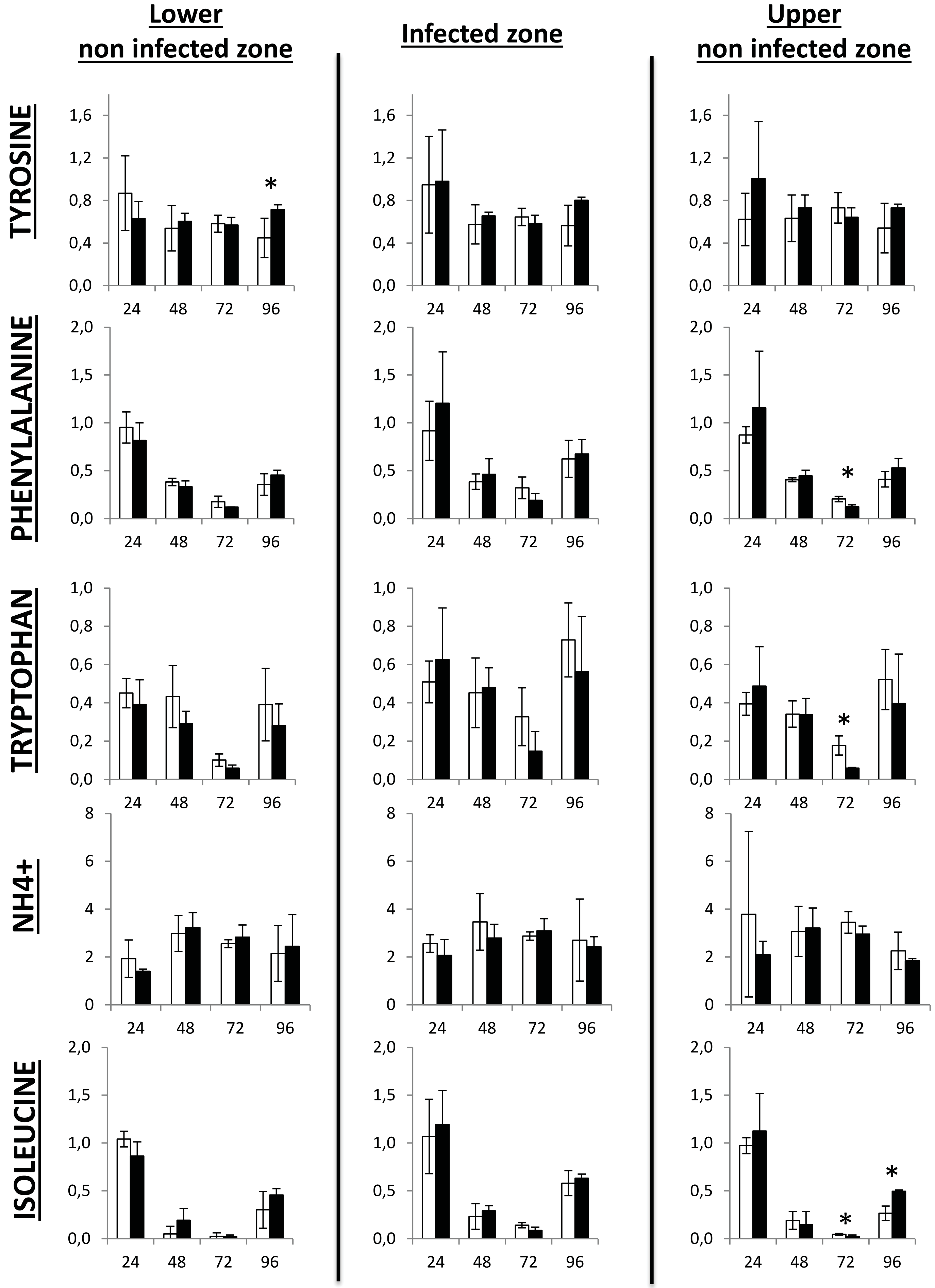
Lower non infected zone
Infected zone
Upper non infected zone
TYROSINE
PHENYLALANINE
TRYPTOPHAN
NH4+
ISOLEUCINE
0,0 0,4 0,8 1,2 1,6
0,0 0,5 1,0 1,5 2,0
0,0 0,2 0,4 0,6 0,8 1,0
0 2 4 6 8
24 48 72 96
*
